# Understanding the Intersection of Race/Ethnicity, Socioeconomic Status, and Geographic Location: A Scoping Review of U.S. Consumer Food Purchasing

**DOI:** 10.3390/ijerph17207677

**Published:** 2020-10-21

**Authors:** Chelsea R. Singleton, Megan Winkler, Bailey Houghtaling, Oluwafikayo S. Adeyemi, Alexandra M. Roehll, JJ Pionke, Elizabeth Anderson Steeves

**Affiliations:** 1 Department of Kinesiology and Community Health, University of Illinois at Urbana-Champaign, Champaign, IL 61820, USA; oadey2@illinois.edu (O.S.A.); aroehll2@illinois.edu (A.M.R.); 2 Division of Epidemiology and Community Health, School of Public Health, University of Minnesota, Minneapolis, MN 55455, USA; mwinkler@umn.edu; 3 School of Nutrition and Food Sciences, Louisiana State University (LSU) & LSU Agricultural Center, Baton Rouge, LA 70803, USA; bhoughtaling@agcenter.lsu.edu; 4 University Library, University of Illinois at Urbana-Champaign, Champaign, IL 61820, USA; pionke@illinois.edu; 5 Department of Nutrition, University of Tennessee, Knoxville, TN 37996, USA; eander24@utk.edu

**Keywords:** intersectionality, food purchasing, diet quality, race, ethnicity, socioeconomic status, urban, rural

## Abstract

Disparities in diet quality persist in the U.S. Examining consumer food purchasing can provide unique insight into the nutritional inequities documented by race/ethnicity, socioeconomic status (SES), and geographic location (i.e., urban vs. rural). There remains limited understanding of how these three factors intersect to influence consumer food purchasing. This study aimed to summarize peer-reviewed scientific studies that provided an intersectional perspective on U.S. consumer food purchasing. Thirty-four studies were examined that presented objectively measured data on purchasing outcomes of interest (e.g., fruits, vegetables, salty snacks, sugar-sweetened beverages, Healthy Eating Index, etc.). All studies were of acceptable or high quality. Only six studies (17.6%) assessed consumer food purchases at the intersection of race/ethnicity, SES, or geographic location. Other studies evaluated racial/ethnic or SES differences in food purchasing or described the food and/or beverage purchases of a targeted population (example: low-income non-Hispanic Black households). No study assessed geographic differences in food or beverage purchases or examined purchases at the intersection of all three factors. Overall, this scoping review highlights the scarcity of literature on the role of intersectionality in consumer food and beverage purchasing and provides recommendations for future studies to grow this important area of research.

## 1. Introduction

Most Americans’ diets fall short of national dietary guidelines [1]. Nearly 75% of Americans consume too few fruits and vegetables, and more than 60% consume excess added sugar, saturated fat, and sodium [2]. Furthermore, most Americans’ overall diet quality is rated moderate to poor [2]. Food purchasing is a critical behavior in shaping the overall nutritional quality of consumed diets [3,4]. Purchases made in full-service (e.g., supercenters, grocery stores, etc.) and limited-service (e.g., corner stores, gas stations, dollar stores, pharmacies, etc.) stores comprise upwards of 63% of an individual’s total daily energy intake [5]; the remaining 37% is acquired from venues such as full-service and fast food restaurants. Additionally, more than 60% of the sugar-sweetened beverages (SSB) and discretionary foods consumed by U.S. adults come from retail food outlets [6]. 

Food retailer availability, adverse dietary behaviors, and the related health consequences are not distributed equally across the U.S. population [7,8,9,10]. Significant inequities in diet and health status have been, and continue to be, documented by race/ethnicity, socioeconomic status (SES), and geographic location (i.e., urban vs. suburban vs. rural) in the U.S. [8,9,10]. However, health disparities are often researched and described by experts in a way that can discount the complex identities of many marginalized individuals [11,12,13]. Intersectionality is a theoretical framework used to describe how multiple social categories measured at the individual level (e.g., race, ethnicity, SES) reflect interlocking systems of privilege and oppression at the societal level [11]. As these realities are experienced jointly, it is important to examine how these factors work together to influence health behaviors such as dietary intake and food purchasing. 

Prior reviews of food and beverage purchasing have primarily focused on evaluating interventions aimed at improving purchasing behaviors [14,15,16,17,18,19], and recently, the use of commercial food purchasing datasets to discover specific purchasing trends [3]. Studies often present information on food and beverage purchasing behaviors at the individual or household-level by racial/ethnic group or SES [3,5]. However, there continues to be a limited synthesized understanding of how the intersectional nature of these factors influences trends in consumer food purchases. Filling this gap in knowledge can inform research and practice approaches to improve food purchasing environments and behaviors among populations with a long-standing history of oppression and marginalization.

Therefore, the primary aim of this scoping review was to identify and summarize scientific studies providing an intersectional perspective on U.S. consumer food purchasing. Specifically, we were interested in assessing food and/or beverage purchasing at the intersection of race/ethnicity, SES, and geographic location as these three factors are often considered in studies of nutritional inequities across populations [8,9,10]. Additional aims of this review included (1) summarize key findings from studies that assessed consumer food purchasing solely by race/ethnicity, SES, or geographic location and (2) identify areas for future research that will expand the field’s understanding of how the intersection of these three factors influences food and beverage purchasing. Thus, findings from this review may significantly contribute to the work of public health researchers, policy makers, and individuals in the private sector seeking to gain a better understanding of food retail, purchasing, and marketing in the U.S. and develop solutions to address nutritional inequities.

## 2. Materials and Methods

### 2.1. Search Strategy and Inclusion Criteria

In December 2019, a systematic search of the literature was conducted to identify peer-reviewed papers on U.S. consumer food and beverage purchasing. A librarian (J. P.) searched the following six databases, selected based on lead sources for peer-reviewed literature among several disciplines including public health, medicine, psychology, sociology, and economics: PubMed, Scopus, PsycINFO, CINAHL, ScoINDEX, and Business Source Ultimate. The search strategy developed by the librarian based on preliminary testing in PubMed (See Supplementary Material Part I) was translated across all remaining databases for optimum article retrieval. All citations returned by the search were extracted and imported into an open-source citation management software. 

The following inclusion criteria were used: (1) published in a peer-reviewed journal, (2) published in 2000 or later (up until December 2019), (3) available in English, (4) based in the U.S., (5) employed an observational study design (e.g., cross-sectional, longitudinal, etc.), (6) analyzed objectively measured food and/or beverage purchasing data collected at any level (i.e., individual, household, or store) from full-service or limited-service stores, and (7) presented findings on purchasing by race/ethnicity, SES, geographic location, or any combination of these three factors. Studies that examined purchasing intersections (i.e., explored interaction terms or reported stratified regression models) for two or more factors were labeled “intersectional”. Studies that presented purchasing findings for a specific intersectional population (example: low-income non-Hispanic Black households living in an urban setting) were also included. These studies were labeled as “targeted”. Since this review aimed to summarize observational data on consumer food purchasing, interventions, natural experiments, and policy evaluation studies were excluded. Furthermore, studies that solely analyzed self-reported food and/or beverage data were also excluded. A wide range of objectively measured purchasing data were considered including store-generated sales data, annotated receipt data, and customer intercept data. Given the large variability in food and beverage purchasing outcomes assessed by selected studies, the types of outcomes considered by the current study were narrowed to a specific list of categories (see *Data Extraction*). 

### 2.2. Study Selection

**Figure 1:**
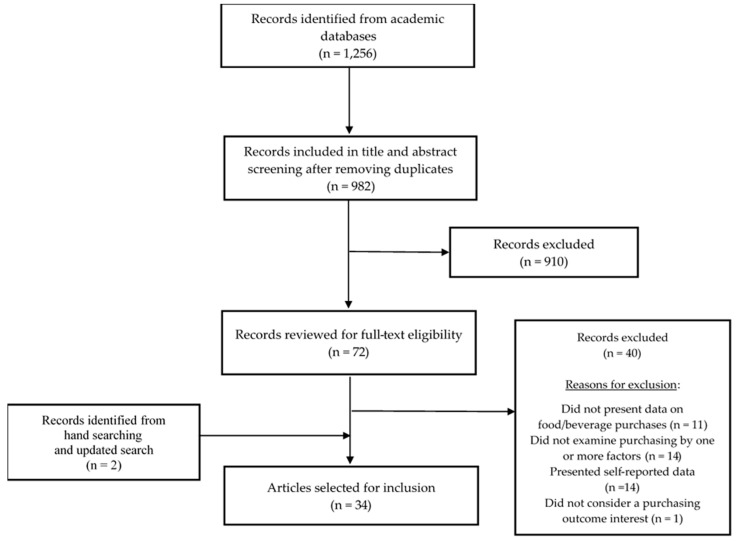
Flow chart for scoping review.

A flow chart describing the study selection process is presented in (Figure 1). The search returned 1256 citations: PubMed (*n* = 430), Scopus (*n* = 354), PsycINFO (*n* = 140), CINAHL (*n* = 181), ScoINDEX (*n* = 28 results), and Business Source Ultimate (*n* = 123). 

After removing duplicate citations, three reviewers (O. S. A., A. M. R., and C. R. S.) reviewed titles and abstracts among 982 unique studies. Titles and abstracts indicated that 910 studies did not meet inclusion criteria. The complete text was retrieved for citations appearing to meet inclusion criteria or were unclear (*n* = 72). Two independent reviewers (O. S. A. and A. M. R.) performed the full text review, and a third reviewer (C. R. S.) made the final decision on inclusion for any disagreements. Excluded studies were ineligible because they (1) did not present findings on food and/beverage purchases (*n* = 11), (2) used self-reported purchasing measures (*n* = 14), (3) did not present findings by one or more of the three factors of interest (*n* = 14), or (4) did not present findings on a purchasing outcome of interest (*n* = 1). Hand searching, specifically forward and backwards reference searching of intersectional papers, was performed to find intersectional studies not captured by the search strategy resulting in the identification of one additional paper. The search was repeated in September 2020 to identify additional intersectional papers published since December 2019. Again, one paper was identified bringing the final number of studies included in this scoping review to 34.

### 2.3. Data Extraction

All authors extracted data from an assigned subset of included studies using a standardized data extraction tool developed by research team members (C. R. S., M. W., B. H., and E. A. S.). Specifically, data on authors, study design, study population, sample size, and detailed information on measurement methods used to capture consumer food and/or beverage purchasing as well as variable definitions for race, ethnicity, SES, and geographic location were extracted. An additional team member performed a quality assessment for each source (see *Methodological Quality Assessment*).

Given the enormous diversity in customer purchasing outcomes examined across the included studies, team members (C. R. S., M. W., B. H., or E. A. S.) extracted food-at-home customer purchasing results for a pre-specified list of product and nutrition outcomes. These particular outcomes were selected because they are often the subject of U.S.-based policy and public health interventions [3,7]: (1) fruits, (2) vegetables, (3) whole grains, (4) salty snacks, (5) desserts, sweet snacks, and candy, (6) sugar-sweetened beverages (SSBs), including regular soda, juice drinks (<100% juice), sports drinks, and energy drinks, (7) non-sugar-sweetened beverages (non-SSBs), including water, diet/zero calorie soda, 100% juice, diet/zero calorie sports drinks, and diet/zero calorie energy drinks, (8) healthy eating index (HEI), (9) total energy (i.e., kilocalories/kcals), (10) specific nutrients, including sugar; saturated fat; and sodium. We extracted results on these outcomes in any form (e.g., weekly expenditures, proportion of weekly purchases, kilocalories/person/day purchased for household, etc.) and prioritized inferential results, although descriptive results were extracted if it was the only data available. Lastly, we extracted results for any study that examined intersections or presented inferential results by race/ethnicity, SES, or geographic location. A narrative format was used to describe review results and identify similarities/differences in population purchasing trends based on intersectionality.

### 2.4. Methodological Quality Assessment

Risk of bias was assessed using the National Heart, Lung, and Blood Institute’s (NHLBI) Quality Assessment Tool for Observational Cohort and Cross-sectional Studies [20]. The tool allowed reviewers to evaluate internal validity across 14 criteria, four of which were deemed not applicable to the studies of consumer food purchasing included in this review (items 6, 7, 10, and 12). One reviewer (O.S.A. or A. M. R.) conducted this assessment, with a second reviewer (C. R. S.) reviewing for agreement. Reviewers recorded yes, no, or cannot determine for each item regarding a study’s original aim/purpose and results. Thus, quality scores represent overall quality of study designs and not necessarily the quality of purchasing results extracted. “Yes” responses were tallied and the highest score a study could receive was a 10. Although the tool was not intended for use as a scoring scheme, we identified scores between 1–4 as low, 5–7 as acceptable, and 8–10 as high quality to assist our results interpretation.

## 3. Results

Thirty-four studies were included in this scoping review [21,22,23,24,25,26,27,28,29,30,31,32,33,34,35,36,37,38,39,40,41,42,43,44,45,46,47,48,49,50,51,52,53,54]. Information on customer purchasing assessment methodologies used across studies is shown in (Table 1). Most studies examined both food and beverage purchasing (*n* = 29; 85.3%) and collected data at the household level (*n* = 24, 70.6%). While several studies assessed purchases from all types of stores (*n* = 24, 70.6%), seven (21.2%) and three (9.1%) studies focused solely on purchasing at limited-service and full-service stores, respectively. A variety of data sources were used across studies with most using Nielsen Consumer Panel data (*n* = 11, 33.3%) or the USDA’s Food Acquisition and Purchasing Survey (FoodAPS) dataset (*n* = 5, 20.8%). Several data collection methods were used to study purchasing including customer intercepts, receipt collection, and Universal Product Code (UPC) scanning. 

Descriptive characteristics of studies are provided in (Table 2). All studies were considered acceptable or high quality according to our interpretation of papers using the NHLBI Quality Assessment Tool for Observational Cohort and Cross-sectional Studies. The majority examined purchasing using a nationally representative sample of U.S. households (*n* = 18, 52.9%). All other studies assessed purchasing locally in a specific city or regionally in the Midwest or Northwest. 

Key findings are described below by intersectional attributes. Studies that presented intersectional results on consumer food and beverage purchases are described first, followed by those that studied a single attribute (i.e., examined purchasing by race/ethnicity, SES, or geographic location alone). Finally, descriptive results from studies with targeted populations are provided.

### 3.1. Intersectional Results 

Key findings from studies that assessed consumer food and/or beverage purchases at the intersection of race/ethnicity, SES, or geographic location are in (Table 3). Details on how each study measured each purchasing outcome of interest are also provided in (Table 3). Only six studies (17.6%) examined any intersection between our three factors of interest [29,34,35,45,47,54]. All six studies examined intersections between race/ethnicity and SES by using interaction terms or stratified regression models. We focused on results with significant interaction terms or with different associative patterns in the stratified models (e.g., association between race/ethnicity and purchasing was significant in opposite directions across SES groups or the association was statistically significant for one SES group and non-significant for the other).

Three studies examined fruit and vegetable purchasing and only one identified different associations between race/ethnicity and purchasing across SES [29,34,45]. Using specific market basket items and stratifying by SES, Palmer et al. (2019) reported more purchasers than non-purchasers of canned/bottled peaches and potatoes among White higher income households (>200% FPL), whereas no significant difference in proportion of purchasers to non-purchasers was observed among White low-income households [45]. In addition, there were significantly fewer purchasers than non-purchasers of potatoes among Black higher income households, which was not observed among Black low-income households [45]. No studies examined whole grain purchasing. Three studies examined salty snacks and desserts, sweet snacks, and candy purchasing [29,34,35], with only one identifying different associations between race/ethnicity and purchasing across stratified SES models [35]. Among households not participating in the Supplemental Nutrition Assistance Program (SNAP), Grummon and Taillie (2018) identified non-Hispanic Black households (henceforth NHB) purchased less salty snacks, desserts, and sweet snacks compared to non-Hispanic White households (henceforth NHW) [35]. In addition, Hispanic households purchased less candy, desserts, and sweet snacks compared to NHW households. These race/ethnicity differences were not observed among SNAP-participating households. Three studies examined SSBs and non-SSBs, but none found significant differences across intersections [29,34,35]. 

One study examined the quality of household food purchases using HEI [54]. However, Vadiveloo et al. (2020) reported no significant interactions between race/ethnicity and family income. Two studies examined overall kilocalories purchased [34,35], with one identifying relevant results [35]. Among SNAP households, Grummon and Taillie (2018) identified that NHB purchased significantly more kilocalories compared to NHW, which was not observed among non-SNAP households. Two studies examined sugar, saturated fat, and sodium, and Grummon and Taillie (2018) reported significant intersectional results for sodium and sugar [34,35]. Hispanics had significantly greater purchasing of sodium compared to NHW among non-SNAP households, which was not observed in SNAP households. In addition, among SNAP households, Hispanics had significantly less purchasing of sugar than NHW, though this was not observed in non-SNAP households. 

Poti et al. (2016) was the only study that examined purchasing outcomes that were not part of our primary outcomes of interest across intersectional attributes [47]. They explored whether household income moderated the association between race/ethnicity and purchasing products with different degrees of processing (e.g., highly processed, minimally processed) and ready-to-eat (e.g., requires cooking, ready-to-heat). Significant interactions between race/ethnicity and SES were identified for basic-processed and requires cooking food purchases. Greater purchasing of both outcomes was observed among NHB and Hispanics compared to NHW among low-income households.

### 3.2. Single Attribute Results

#### 3.2.1. Race/Ethnicity

Fifteen studies (44.1%) examined purchasing outcomes across racial and/or ethnic groups [27,29,30,39,42,43,45,46,47,48,49,50,51,53,54]. All studies examined purchasing among NHW, 14 examined purchasing among NHB, 14 studied purchasing among Hispanic, nine examined purchasing among non-Hispanic Other (or a different author definition that collapsed multiple racial/ethnic groups), and three investigated purchasing among Asian (using the author definition). Key findings from studies that presented racial/ethnic differences in consumer food and/or beverage purchases are described in detail in Supplementary Material Part II (Table S1).

#### 3.2.2. Socioeconomic Status

We identified 19 (55.9%) studies that examined purchasing outcomes across SES categories [26,27,29,30,31,32,33,34,36,37,39,42,43,45,49,50,51,52,54]. Ten studies evaluated SES by looking across household income levels, while seven studies used federal food assistance program participation status (i.e., SNAP status), four studies used education level, one study used employment status, and one study classified food retail stores based on income of the surrounding neighborhoods. In three studies, SES was examined in more than one way (e.g., both income and education levels were assessed). Supplementary Material Part II (Table S2) present key findings from the studies that evaluated SES differences in consumer food and/or beverage purchases. 

#### 3.2.3. Geographic Location

We did not identify any studies that examined differences in customer food and/or purchasing by geographic setting (i.e., urban vs. suburban vs. rural).

### 3.3. Targeted Population Results

#### 3.3.1. Intersectional Targeted Populations

Eleven studies (33.3%) were labeled targeted [21,22,23,24,25,26,28,38,40,41,44]. Five examined consumer food purchases among an intersectional targeted population [23,26,38,40,44]. These populations were low-income individuals or households living in an urban city [23,38,40,44] and NHBs living in an urban city [26]. All studies with a low-income urban population focused solely on limited-service store purchasing. Three studies assessed fruit and vegetable purchasing while none examined whole grain purchasing [26,40,44]. Overall, fruit and vegetable purchasing was moderate to low. Chrisinger et al. (2018) reported that 14% of total food expenditures among a small sample of NHB women were spent on fruits and vegetables [26]. Lent et al. (2014) and O’Malley et al. (2013) found that fruits and vegetables comprised 2.3% and 5% of purchases from limited-service store shoppers in low-income urban communities, respectively [40,44]. All five studies examined purchasing of salty snacks, desserts, sweet snacks, and/or candy. While Chrisinger et al. (2018) reported that these items represented only 11% of food expenditures among NHB women, the other four articles found that these items represented a large percentage of customer purchases in limited-service stores (>20%). All five studies assessed SSB purchasing; only three assessed non-sweetened beverage purchasing [23,40,44]. SSB were the items most often purchased across all studies. No study examined the quality of purchases using HEI. Only Borradaile et al. (2009) and Lent et al. (2014) examined kilocalories, saturated fat, sugar, and sodium content of purchases [40,44]. Both studies reported high volumes of each nutrient among customer purchases from limited-service stores. Key findings from studies that assessed consumer food and/beverage purchases with a targeted population are reported in (Table S3) of Supplementary Material Part III.

#### 3.3.2. Single Factor Targeted Populations

The remaining six targeted studies reported purchasing for a single factor targeted population [21,22,24,25,28,41] including low-income individuals or households [21,41] and individuals or households residing in an urban city [22,24,25,28]. Low-income targeted populations focused on participants of federal food assistance programs such as SNAP and the Special Supplemental Nutrition Program for Women, Infants, and Children (WIC). Key findings from studies that focused on single factor targeted populations are also presented in Supplementary Material Part III (Table S3).

## 4. Discussion and Future Directions

We aimed to summarize peer-reviewed scientific studies that assessed U.S. food and/or beverage purchasing at the intersection of race/ethnicity, SES, and geographic location, and recommend future approaches to expand this area of research. Food purchasing behaviors have been reviewed previously [4,14,15,16,17,18,19], although this scoping review is the first to (1) synthesize findings on food and beverage purchases by race/ethnicity, SES, and geographic location and (2) examine the intersectional nature of these factors. Our main finding is a limited number of studies published since 2000 provide an intersectional perspective on food and/or beverage purchasing across our three factors of interest, which have been consistently linked with diet and health inequities [29,34,35,45,47,54]. Thus, the vast majority of studies evaluated purchasing by a single attribute or within a specific targeted population. Below, we describe the implications of our review findings by attribute and provide future recommendations for studies seeking to contribute to this literature. A comprehensive list of future directions is provided in (Table 4).

### 4.1. Understanding the Intersection of Race/Ethnicity, SES, and Geographic Location

As mentioned, several studies have reported health and nutritional inequities by race/ethnicity, SES, and urban vs. rural status [8,9,10]. Assessing the intersectional nature of these factors may provide researchers new insight into food and beverage purchasing patterns to inform the design of policy, systems, and environmental change interventions that advance health equity [11,12,13]. Only six studies (17.6%) in this review examined the intersection of two attributes with all assessing race/ethnicity by SES differences [29,34,35,45,47,54]. Given the small number of studies and the inconsistency in food and beverage purchasing outcomes considered, specific patterns in purchasing could not be identified. Thus, we still have limited understanding of how measures reflecting SES moderate racial/ethnic differences in food purchasing. Future studies should examine U.S. consumer food and/or beverage purchases at the intersection of more than two factors. Since none of the intersectional studies considered geographic location, future studies should determine how urban vs. suburban vs. rural status moderates racial/ethnic and SES differences in purchasing. Moreover, since most studies included in this review (*n* = 18, 52.9%) examined purchasing using data collected from a nationally-representative sample of U.S. households, future studies could focus on providing an intersectional perspective on food and beverage purchasing at the local and regional levels, especially in the South and West regions of the country. 

### 4.2. Race/Ethnicity

Several reviewed studies (*n* = 15, 44.1%) presented purchasing findings by race/ethnicity [27,29,30,39,42,43,46,47,48,49,50,51,53,54]. Despite the large number of studies conducted to date, inconsistencies exist. Across studies, we identified more consistent patterns between NHW and Hispanics regarding purchasing, with Hispanics exhibiting healthier purchasing patterns relative to NHW. For example, we found that most studies examining differences between NHW and Hispanics reported greater fruit and/or vegetable purchasing and less salty snack, dessert, and candy purchasing. Fewer consistencies were noted between NHB and NHW although several studies reported greater SSB and sugar purchasing among NHB compared to NHW. These findings align with the dietary consumption literature, which continues to highlight significant racial/ethnic differences in intake among adults and children [8,10,54,55,56]. Additional studies are needed to establish consistent patterns in food and beverage purchasing by racial/ethnic group. Future studies should evaluate consumer food and/or beverage purchases across a greater variety of racial/ethnic groups (i.e., non-Hispanic Asian, Native American, Pacific Islander, etc.). Given the heterogeneous composition of all races and ethnicities, future studies could conduct robust assessments of purchasing within groups, which will permit the study of characteristics such as acculturation and nativity—two factors that are often considered in studies of diet quality [55,56]. In recent years, public health research has placed greater emphasis on socio-political factors that create racial/ethnic inequities in health such as structural and systemic racism [57]. Future studies should consider how these important social factors impact food and beverage purchasing.

### 4.3. Socioeconomic Status

Most studies included in this review (*n* = 19, 55.9%) examined SES differences in consumer food and/or beverage purchases [26,27,29,30,31,32,33,34,36,37,39,42,43,45,49,50,51,52,54]. These findings underscore that identifying purchasing patterns by SES continues to be a major priority in the field; included studies generally showed a lower likelihood of fruit, vegetable, and whole grain purchases and a higher likelihood for discretionary product purchases (i.e., salty snacks, sweets, and SSB) among consumers with lower incomes compared to higher incomes [58]. Low-income consumers have been described as more likely to be targeted by marketing for food items high in kilocalories, saturated fat, added sugars, and sodium in retail food outlets [59,60,61], and the results of this review and reviews of diet quality differences by SES align with such observations given the poor quality of food and beverage purchases observed [62]. Furthermore, qualitative evidence has found that low-income consumers are more likely than consumers with higher incomes to purchase less costly, energy-dense and nutrient-poor products amid household financial constraints [63]. Approaches are needed to assess SES differences in purchasing using intersectional theory as a guiding framework to discern opportunities for tailored policy, systems, and environmental change interventions to improve the dietary quality of populations who experience diet-related health disparities [11]. Moreover, given the increase in studies that have evaluated the public health implications of community-level factors such as economic deprivation, blight, and gentrification displacement, future studies should also consider these factors in the context of consumer food and/or beverage purchasing [64,65].

### 4.4. Geographic Location

No studies included in this review examined geographic differences (i.e., urban vs. suburban vs. rural) regarding consumer food and/or beverage purchasing. This is particularly concerning because rural populations experience a higher burden of major diet-related diseases than urban populations (e.g., heart disease, cancer, stroke), which represent the leading causes of death in the U.S. [66]. The idea that more food environment research specific to rural people and places is needed is not new [67,68]. Rural residents have been shown to have few opportunities for choosing food and beverage options aligned with dietary guidelines in general when compared to residents of more urban areas [69,70]. It is unknown how food environment disparities influence differences in purchasing and dietary patterns between urban, suburban, rural populations, and how multiple socio-demographic factors such as race/ethnicity and SES to influence food purchasing disparities. This requires much more focus moving forward, in order to mitigate prominent health disparities in the U.S. 

### 4.5. Targeted Populations

We included studies that targeted a specific population in order to provide greater context to findings from studies that evaluated consumer food and/or beverage purchases by race/ethnicity, SES, or geographic location [21,22,23,24,25,26,28,38,40,41,44]. While several studies were labeled targeted (*n* = 11, 33.3%), the variety of target populations considered was limited to primarily low-income individuals and households residing in an urban setting. No targeted study described purchasing in a rural population or specific racial/ethnic group that is often understudied in this area of research: non-Hispanic Asian, Native American, etc. Thus, studies are needed to address this gap and contribute more knowledge on the food and beverage purchases of intersectional target populations that represent two or more attributes (example: low-income Hispanic families living in a rural setting).

### 4.6. Limitations

Several limitations should be considered alongside review results. First, like most reviews, there were limitations in the research strategy. While a trained research librarian (J.P.) guided the literature search process, we limited our search to six databases with a set combination of key words. There is the possibility that relevant studies available in other databases were not included in this review. The large variety of purchasing measures presented by included studies made it not feasible to extract all of the purchasing data. Data from included studies were extracted based upon pre-selected purchasing outcomes of interest such as food groups (fruits, vegetable, whole grains, etc.) and nutritional characteristics (HEI, kcals, etc.). Thus, some purchasing outcomes (e.g., meat, dairy products, etc.) were not examined because they fell outside the scope of our data extraction protocol. An “Other” category was included to allow for the extraction of specific results of interest (example: nutrient claims) that did not align with the pre-specified categories. 

Interventions and natural experiments that aimed to modify food and/or beverage purchasing were excluded from the review. It is possible that baseline findings from these studies documented food and beverage purchasing by one or more of our factors of interest. Because this scoping review solely focused on U.S. consumer food and beverage purchasing, findings may not be generalizable to other countries. The methodological assessment tool was not designed to assess the quality of nutrition studies or studies of consumer food purchasing. As previously mentioned, quality scores reported in (Table 2) solely reflect study design and not the quality of the purchasing data presented in the paper. Finally, during the data extraction phase, statistical significance was relied on heavily to identify which results to include in this review. While this made data extraction practical for the research team, this method limits the ability to account for the magnitude of differences in the various analyses. Detailed descriptions of key findings from included studies presented in this paper and the supplemental tables allow the reader to explore consumer purchasing outcomes in more detail.

## 5. Conclusions

This scoping review found that few studies to date have examined consumer food and beverage purchasing in the U.S. at the intersection of race/ethnicity, SES, and geographic location, despite the large number of studies that assessed purchasing by one of these factors alone. To expand this area of research, future studies should use intersectional theory to guide efforts to evaluate consumer food and/or beverage purchasing in the U.S. at the intersection of race/ethnicity, SES, and geographic location rather than continuing to examine factors individually. Furthermore, future studies should select data collection and assessment methodologies that allow for the gathering of rich data on the relationship between intersectional identity and food purchasing [13]. For example, consumer purchasing intercepts coupled with qualitative interviews that elicit rich descriptions of factors influencing dietary purchasing decisions may be a useful approach to increase our knowledge base on the socio-political and cultural factors that create persistent inequities in food purchasing behavior, dietary intake, and health.

## Figures and Tables

**Table 1:** Summary of Customer Purchasing Data Assessment Methodologies of Included Studies (*n* = 34).

Items Assessed	Purchasing Level	Retail Stores	Data Type	Data Source	Data Collection Method ^a^
Beverages only (2) Foods only (3) Foods and Beverages (29)	Individual (8) Household (24) Store (2)	Full-service only (3) Limited-service only (7) All Stores (24)	Primary data collection (14) Secondary data analysis (19) Primary and secondary data (1)	Nielsen Consumer Panel (11) USDA FoodAPS (5) The STORE Study (3) The SHOPPER Study (3) National Food Stamp Program Survey (1) IRI Consumer Network Panel (1) Consumer Expenditure Survey (1) Other (9)	Retailer-scanner data (3) Customer store intercepts (7) Customers scanned UPCs (11) Customer receipt collection (5) Multiple methods/Other (8)

Note. USDA, United States Department of Agriculture; FoodAPS, Food Acquisition and Purchasing Survey; STORE, the Staple Food Ordinance Evaluation; SHOPPER, the Study of Household Purchasing Patterns, Eating, and Recreation; UPC, Universal Product Code. ^a^ Primary method used to collect information on purchases. Studies using multiple methods (e.g., receipt collection and barcode scanning) were categorized as multiple methods and one study used detailed diaries, which was categorized as other.

**Table 2:** Descriptive Characteristics of Included Studies (*n* = 34).

Author, Year	Study Purpose	Study Year(s) ^a^	Study Location	Sample Size	Intersectional Attribute(s)	Sample Demographics ^b^	Stores Assessed	QA ^c^
Andreyeva, 2012	Describe supermarket beverage purchases of WIC and SNAP households.	2011	New England	39,172 Households	Targeted: Low-Income	100% WIC Participation, 54% SNAP Participation	Full-Service	6
Appelhans, 2017	Determine if household food purchases predict diet quality and nutrient density.	2014–2016	Chicago, IL	196 Households	Targeted: Urban	Mean age: 44; 83% female; 31% (NHW), 44% (NHB), 11% (Hisp), 13% (NHO); 38% (PIR: 0–1.99), 29% (2–3.99), 16% (4–5.99), 18% (≥ 6)	All Stores	9
Borradaile, 2009	Describe after-school corner stores purchases of low-income children.	2008	Philadelphia, PA	833 Shoppers	Targeted: Low-Income + Urban	Grade range: 4–6 grade; 54% (NHW), 11.6% (NHB), 22.9% (Hisp), 10.8% (NHA); 82.1% of students at participating schools eligible for free/reduced lunch.	Limited-Service	5
Caspi, 2017 [1]	Examine differences in food and beverage purchases by type of limited-service store.	2014	Minneapolis, MN	661 Shoppers	Targeted: Urban	47% (NHW), 34% (NHB), 3% (Hisp), 3% (NHA), 3% (NHO); 38% ≤ high school diploma	Limited-Service	7
Caspi, 2017 [2]	Determine if food and beverage purchases at limited-service stores with health-promoting features are healthier.	2014	Minneapolis, MN	594 Shoppers	Targeted: Urban	Mean age: 40; 58% male; 48% (NHW), 36% (NHB), 3% (Hisp), 3% (NHA), 3% (NHO); 36% ≤ high school diploma	Limited-Service	9
Chrisinger, 2018 [1]	Compare high-calories and low-calorie food purchases of Black women by store type.	2012	Philadelphia, PA	35 Shoppers	Targeted: Black + Urban ^d^	Mean age: 39; 100% female; 100% Black Identifying; 37% Annual Income ≤ FPL	All Stores	8
Chrisinger, 2018 [2]	Assess the healthfulness of household food purchases by SNAP and WIC participation status.	2012–2013	National	4962 Households	RE, SES	17.2% (30–39 years), 18.5% (40–49), 20.2% (50–59), 29.9% (60+); 64% female; 70% (NHW), 10.2% (NHB), 13.7% (Hisp), 6% (NHO); 13.1% (SNAP participant), 19.3% (SNAP-Eligible Non-Participant), 67.6% (Ineligible Non-Participant)	Full-Service	8
Crane, 2019	Identify gender differences in the nutrient quality of food purchases.	2014–2016	Midwest	202 Households	Targeted: Urban	29.9% (NHW), 45.6% (NHB), 5.9% (Hisp), 18.6% (NHO); 40.6% receive government food assistance benefits	All Stores	8
Cullen, 2007	Characterize food purchases of households by educational level and ethnicity.	2004	Houston, TX	167 Households	RE x SES	45.8% (<40 years); 74.8% (female); 11.2% (NHW), 41.1% (NHB), 39.3% (Hisp), 2.8% (NHO); 46.7% (≤ High School Graduate), 28% (Some College), 14% (College Graduate), 6.5% (Advanced Degree)	All Stores	8
Ford, 2014	Examine trends in purchases of consumer packaged goods among households with children age 2–5 years old.	2000–2011	National	14,110 Households	RE, SES	68.3% (NHW), 10.3% (NHB), 16.8% (Hisp), 4.8% (NHO); 17.3% (<131% FPL), 14% (131–185% FPL), 68.3% (> 185% FPL)	All Stores	7
Frankle, 2017	Describe differences in the purchasing of SNAP-eligible foods by SNAP participation status.	2012–2014	New York, New England	188 Stores	SES	NR	Full-Service	7
French, 2019	Assess differences in the nutritional quality of foods and beverages purchased by household income level.	2014–2016	Chicago, IL	202 Households	SES	15.3% (18–24 years), 47.5% (30–49), 36.6% (50+); 83% (female); 29.7% (NHW), 43.1% (NHB), 24.7% (Hisp); 24.3% (PIR: 0–1.3), 38.6% (PIR: 1.4–3.4), 37.1 (3.5+)	All Stores	7
Gorski Finding, 2018	Determine if neighborhood retail food access is associated with overweight/obesity in children.	2012–2013	National	3748 Children	SES	SNAP Participants: 32% (NHW), 31.6% (H), 29.7% (NHB), 6.7% (O); SNAP-Eligible Non-Participants: 33.5% (NHW), 41.2% (Hisp), 19.6% (NHB), 5.7% (NHO); Ineligible Non-Participants: 65.0% (NHW), 16.9% (Hisp), 9.8% (NHB), 8.3% (NHO)	All Stores	8
Grummon, 2017	Examine the nutritional profile of household food and beverage purchases by SNAP participation status.	2012–2013	National	70,477 Households	RE x SES ^e^	SNAP Participants: Mean age: 55.5, 77% (NHW), 14% (NHB), 5% (Hisp), 4% (NHO); Income-Eligible Non-Participants: Mean age: 59.1, 82% (NHW), 8% (NHB), 4% (Hisp), 6% (NHO); Higher Income Non-Participants: Mean age: 59.3, 83% (NHW), 8% (NHW), 4% (Hisp), 5% (NHO).	All Stores	8
Grummon, 2018	Describe differences in the unhealthy food and beverage purchases by race/ethnicity and SNAP participation status.	2010–2014	National	30,403 Households	RE x SES	Mean age: 59.2; 87% (NHW), 8% (NHB), 5% (Hisp); 17.5% SNAP Participations; 16% (SNAP among NHW), 27% (SNAP among NHB), 21% (SNAP among Hisp)	All Stores	7
Gustafson, 2017	Determine how neighborhood food store availability influences food stores choice and food store purchases.	2012–2013	National	2962 Households	SES	53% (SNAP Participants); 47% (SNAP-Eligible Non-Participants)	All Stores	6
Jones, 2003	Assess differences in food shopping behaviors and consumption patterns between grocery store customers in low-income and high-income areas.	2001	Columbus, OH	6 Stores	SES	Low-Income Areas: 76.2% (NHW), 21.7% (NHB), 2.0% (NHO); High-Income Areas: 93.6% (NHW), 3.5% (NHB), 3.0% (NHO)	Full-Service	6
Kiszko, 2015	Describe the food and beverage purchases of bodega shoppers in low-income communities.	2012	New York City	779 Shoppers	Targeted: Low-Income + Urban	Mean age: 39.1; 51.5% female; 57.0% (Hisp), 34.9% (NHB), 8.1% (NHO); 53% of shoppers had an annual income ≤ USD 25,000	Limited-Service	5
Lenk, 2018	Assess associations between customer characteristics, shopping patterns, and the healthfulness of purchases in limited-service stores.	2014	Minneapolis, MN	661 Shoppers	Targeted: Urban	47% (NHW), 36% (NHB), 17% (NHO); 38% ≤ high school, 37% (some college), 26% (≥college degree)	Limited-Service	6
Lent, 2014	Describe corner store purchases by age group in a low-income urban neighborhood.	2011	Philadelphia, PA	9283 Shoppers	Targeted: Low-Income + Urban	75.5% adults, 15.5% adolescents, 9.9% children; 41.4% female.	Limited-Service	6
Lin, 2014	Examine the roles of food prices and supermarket accessibility in determining food purchases of low-income households.	1996–1997	National	882 Households	Targeted: Low-Income	100% SNAP Households	All Stores	8
Ng, 2016	Evaluate racial/ethnic and income trends in calories purchased in households with children.	2000–2013	National	64,709 Households	RE, SES	NR	All Stores	7
Ng, 2017	Estimate trends in added sugars in beverage purchases among US households by race/ethnicity and socioeconomic status.	2007–2012	National	110,539 Households	RE, SES	NR	All Stores	8
O’Malley, 2013	Determine the feasibility of increasing fruit and vegetable offerings in corner stores.	NR	New Orleans, LA	60 Shoppers	Targeted: Low-Income	48.3% female; 88.3% (AA); 63.3% Annual Income < USD 25,000	Limited-Service	6
Palmer, 2019	Explore food store selection and food purchases in the Northeast using 3 different data sources.	2012–2014	Northeast	IRI CNP: 12,770 Households CES: 3428 Households	SES	IRI Consumer Network Panel (CNP) data: 19.4% (low income, 80.6% (non-low income); Consumer Expenditure Survey (CES) data: 10% of households on SNAP	All Stores	7
Paulin, 2001	Compare food expenditure patterns of Hispanics to Non-Hispanics.	1995–1996	National	13,367 Households	RE	9.2% Hispanic Households, 90.8% Non-Hispanic Households	All Stores	8
Poti, 2016	Examine associations between race/ethnicity, ready-to-eat, highly-processed food and beverage purchasing.	2000–2012	National	157,142 Households	RE x SES	81.3% (NHW), 9.3% (NHB), 7.1% (Hisp)	All Stores	7
Stern, 2016	Determine if food store selection is associated with the nutrient profile of package food purchases across racial/ethnic groups	2007–2012	National	356,611 Households	RE	81.8% (NHW), 8.7% (NHB, 5.1% (Hisp), 4.2% (NHO); 19.0% (≤185% FPL), 43.0% (185–400% FPL), 38% (≥400% FPL)	All Stores	7
Taillie, 2016	Assess the relationship between food retail chain type and the healthfulness of food purchases.	2000–2013	National	164,315 Households	RE, SES	81% (NHW), 9% (NHB), 5% (Hisp), 4% (NHO); 10% of households ≤ 130% FPL	All Stores	7
Taillie, 2017 [1]	Describe the prevalence of price promotions among food and beverage purchases of households with children.	2008–2012	National	90,046,893 Purchases	RE, SES	NR	All Stores	6
Taillie, 2017 [2]	Examine trends in the proportion of packaged food and beverage purchases with a low-nutrient or no-nutrient claim.	2008–2012	National	80,038,247 Purchases	RE, SES	NR	All Stores	7
Taillie, 2018	Compare the nutritional profile of food and beverages of SNAP participants to non-participants.	2010–2014	National	76,458 Households	SES	SNAP Participants: Mean age: 54.5, 76.5% (NHW), 13.8% (NHB), 5.7% (Hisp), 4.0% (NHO); Income-Eligible Non-Participants: Mean age: 58.4, 82.0% (NHW), 8.3% (NHB), 4.5% (Hisp), 5.3% (NHO); Higher-Income Non-Participants: Mean age: 58.5, 82.9% (NHW), 7.9% (NHB), 4.4% (Hisp), 4.7% (NHO)	All Stores	7
Vadiveloo, 2019	Describe geographic differences in the diet quality of household food purchases.	2012–2013	National	3961 Households	RE	Mean age: 50.6; 70.2% female; 70.3% (NHW), 9.9% (NHB), 13.0% (Hisp), 6.8% (NHO); 16.9% (FPL<130%), 41.1% (130–349%), 42.0% (≥350%); 34.6% rural households	All Stores	7
Vadiveloo, 2020	Evaluate racial/ethnic, socioeconomic, and weight-based differences in the diet quality of household food purchases.	2012–2013	National	3961 Households	RE x SES	Mean age: 50.6; 70.2% female; 70.3% (NHW), 9.9% (NHB), 13.0% (Hisp), 6.8% (NHO); 16.9% (FPL<130%), 41.1% (130–349%), 42.0% (≥350%); 57.8% high degree/some college; 12.7% SNAP participation; 34.6% rural households	All Stores	8

Note: AA, African American; FPL, Federal poverty limit; Hisp, Hispanic; NHA, non-Hispanic Asian; NHB, non-Hispanic Black; NHW, non-Hispanic White; NR, None Reported; NHO, non-Hispanic Other (according to the authors’ definition); PIR, Poverty-to-Income ratio; QA, Quality Assessment; RE, Racial/ethnic differences; SES, Socioeconomic differences; SNAP, Supplemental Nutrition Assistance Program; WIC, Special Supplemental Nutrition Program for Women, Infants, and Children. ^a^ Study year (s) reflect the year the data was collected. If data collection dates were not provided, the date the statistical analysis was performed was recorded. ^b^ Demographic information on race/ethnicity, socioeconomic status, and urban/rural status are provided in the table. If socioeconomic information was not available, descriptive statistics for education level or employment status were recorded (if provided by authors). ^c^ The National Heart, Lung, and Blood Institute’s (NHLBI) quality assessment tool for observational cohort and cross-sectional studies was used for quality assessment: https://www.nhlbi.nih.gov/health-topics/study-quality-assessment-tools. ^d^ This targeted study also assessed SES differences. ^e^ “X” indicates that intersectional information is provided on the two factors listed.

**Table 3:** Key Findings from Intersectional Studies (*n* = 6).

Authors (Year)	Intersection Groups	Purchasing Outcomes Examined										Key Findings ‡
		F&V	WG	SS	Dess.	SSB	Bev	HEI	Kcals	Nutri.	Other	
Cullen (2007)	Race x SES	X		X	X	X	X					Interactions between ethnicity of participant (Hisp versus non-Hispanic [NHW and NHB combined]) and SES (highest education of household: high school graduate or less versus some college or more) were explored. No significant interactions were identified for purchasing (percent of total grocery dollar spent on category) of fruit, vegetables, salty snacks, cakes/pies/desserts, candy, carbonated and sweetened drinks, 100% fruit juice, and water.
Grummon (2017)	Race x SES	X		X	X	X	X		X	X		Interactions between race/ethnicity of the head of household (NHW, Hisp, NHB, NHO) and SES (SNAP participant, income-eligible nonparticipant, higher income nonparticipant) were explored. After adjusting for multiple comparisons, no significant interactions were identified for purchasing (kcal/capita/day) of fruit, vegetables, salty snacks, desserts and sweet snacks, candy and gum, SSBs, 100% juice, total energy, sugar, saturated fat, and sodium.
Grummon (2018)	Race x SES			X	X	X			X	X		Differences by race/ethnicity (NHW, NHB, Hisp) tested in models stratified by SES (SNAP participant v. non-participant with household income <250% FPL). Significant race/ethnicity differences varied across SES: Among non-participants and comparing to NHW (ref), NHB had significantly less purchasing (kcals/capita/day) of desserts and sweet snacks and salty snacks and Hisp had less purchasing of desserts and sweet snacks and candy but more purchasing of sodium (mg/capita/day); no significant differences by race/ethnicity occurred for these outcomes among SNAP participants. Among SNAP participants and comparing to NHW, NHB had more purchasing of overall kcals and Hisp less purchasing of sugar (g/capita/day); no significant differences by race/ethnicity occurred for these outcomes among non-participants. Remaining outcomes (SSBs and saturated fat) either did not have significant differences across race/ethnicity or significant differences by race/ethnicity were in the same direction across SES groups.
Palmer (2019)	Race x SES	X										Proportion of purchasers compared to non-purchasers for specific market basket items examined across SES (household income <200% FPL [low] v. > 200% FPL [high]) and race (White, Black) and ethnicity (Hispanic) groups. Among White high income, there were significantly more purchasers than non-purchasers of canned/bottled peaches and potatoes; no significant differences identified among White low income. Among Black high income, there were significantly fewer purchasers than non-purchasers for potatoes; no significant difference identified among Black low income. Remaining outcomes (frozen broccoli) and groups (e.g., Hisp of low or high income) either did not have significant differences or were in the same direction across SES groups.
Poti (2016)	Race x SES										X	Interactions between race/ethnicity (NHW, Hisp, NHB) and SES (household income: <USD 25,000 [low], USD 25,000–USD 49,999, USD 50,000–USD 74,999 and > USD 75,000 [high]) were tested for other outcomes: Proportion of purchases (% of kcals) by 4 categories of degree of processing (minimally-, basic-, moderately- and highly-processed [HP]) and 3 categories of ready-to-eat (requires cooking, ready-to-heat, ready-to-eat [RTE]). Small, though significant, differences identified for basic-processed and requires cooking. Basic-processed *food-only purchases*: NHB and Hisp had greater purchasing than NHW at low-income; at high income, differences narrowed and purchasing was more similar across groups. Requires-cooking *food-only purchases*: NHB and Hisp greater purchasing than NHW at low-income; at high income, differences narrowed and purchasing was similar across groups. No other significant interactions reported.
Vadiveloo (2020)	Race x SES							X				Interactions between race/ethnicity of primary respondent (NHW, NHB, Hisp, NHO) and family SES (<130% of FPL, 130–349% ≥350%) were explored. No significant interaction was identified for the overall quality of food-at-home purchases as measured by HEI-2015 total score.

Note: SES, socioeconomic status; NHW, non-Hispanic White; NHB, non-Hispanic Black; Hisp, Hispanic; NHO, non-Hispanic Other following author definition; SNAP, Supplemental Nutrition Assistance Program; FPL, Federal poverty limit; F&V, fruits and/or vegetables; WG, whole grains; SS, Salty Snacks; Dess., desserts, sweet snacks and candy; SSB, sugar-sweetened beverages; Bev, non-sweetened beverages; HEI, healthy eating index; Kcals, kilocalories; Nutri., sugar, saturated fat, and/or sodium; Other, other purchasing outcomes of interest; ref, reference group in modeling; HP, highly-processed; RTE, ready-to-eat; g, grams; mg, milligrams. ‡ Findings present results from adjusted models unless otherwise noted. Significant results follow the authors’ definition (e.g., some use Bonferroni correction). Underline-bold highlights purchasing outcomes of interest in this review. *Underline-italics* indicates when results for kilocalories/energy density, sugar, saturated fat, sodium, or other category was examined among food purchases and beverage purchases separately.

**Table 4:** Recommendations for Future Directions in Assessing U.S. Consumer Food and Beverage Purchasing.

Intersectional Attribute:	Future Directions:
General	• Compare food and beverage purchasing patterns among full-service and limited-service stores across racial/ethnic groups, SES, and urban/rural status. Specificity regarding purchasing decisions by store type within these broad categories is recommended to inform tailored public health interventions.
Two or More Factors: Race/Ethnicity, SES, and Geographic Location	• Prioritize examining U.S. consumer food and/or beverage purchases at the intersection of two or more factors (i.e., race/ethnicity, SES, and geographic location). • Determine how urban/rural status moderates racial/ethnic and SES differences in food and beverage purchasing.
Race/Ethnicity	• Prioritize evaluating consumer food and/or beverage purchases across a greater diversity of racial/ethnic groups: NHB, Hispanic, Asian, Native American, Pacific Islander, etc. • Examine heterogeneity of purchasing within racial and ethnic groups (example: Hispanic subcultures). • Move beyond assessing “race” as a risk factor and determine how systemic and structural racism influences food and beverage purchasing.
SES	• Consider SES differences in purchasing for food and beverage groups/items that are understudied (i.e., whole grains, non-sweetened beverages) • Assess the relationship between purchasing and community-level factors such as economic deprivation, gentrification displacement, crime, and blight.
Geographic Location Urban vs. Rural	• Examine U.S. consumer food and/or beverage purchases by geographic location at the national, regional, and local levels. • Evaluate urban vs. suburban vs. rural purchasing patterns by store type: full-service vs. limited service. • Prioritize perspectives from minority populations in rural areas regarding influences on food and beverage purchasing.
Targeted Populations	• Study consumer food and/or beverage purchasing among single factor targeted populations that represent populations beyond low-income and/or urban. • Assess consumer food and/or beverage purchasing among intersectional targeted populations that represent 2+ attributes (example: low-income Hispanic families living in a rural area).

Note. SES, Socioeconomic Status; NHW, non-Hispanic White; NHB, non-Hispanic Black.

## Supplementary Materials

The following are available online at https://www.mdpi.com/1660-4601/17/20/7677/s1, Supplementary Material Part I: Search Terms, Supplementary Material Part II: L Single Attribute Results, Table S1: Key Findings from Studies Examining Racial/Ethnic Differences, Table S2: Key Findings from Studies Examining Socioeconomic Differences, Supplementary Material Part III: Targeted Results, Table S3: Key Findings from Targeted Studies.
