# Supplementary material for: Understanding the Intersection of Race/Ethnicity, Socioeconomic Status, and Geographic Location: A Scoping Review of U.S. Consumer Food Purchasing
Source: Int J Environ Res Public Health. 2020 Oct 21;17(20):7677. doi: 10.3390/ijerph17207677 (PMC7593902; doi:10.3390/ijerph17207677)
Supplement: Supplementary file 1 [file ijerph-17-07677-s001.pdf]

SUPPLEMENTARY MATERIAL

Table of Contents

Part I – Search Terms..2

Part II – Single Attribute Results...3

Race/ethnicity...3

Socioeconomic Status...5

Table S1. Key Findings from Studies Examining Racial/Ethnic Differences.........8

Table S2. Key Findings from Studies Examining Socioeconomic Differences....13

Part III – Targeted Results...19

Single Factor Targeted Populations...19

Table S3. Key Findings from Targeted Studies..21

References……………………………………………………………..……………………25

PART I

Search Terms

(“socioeconomic status” OR “socioeconomic factors”[mh] OR ethnicity OR “ethnic groups”[mh] OR “food assistance”[mh] OR “food stamps” OR “low income” OR “poverty”[mh] OR “nutrition assistance” OR “racial disparity” OR “ethnic disparity” OR “SNAP” OR “supplemental nutrition assistance program” OR urban OR rural OR suburban OR “urban population”[mh]) AND (“food purchas” OR “consumer purchas*” OR “consumer behavior”[mh] OR “consumer packaged goods” OR “store bought food” OR “store bought beverage” OR “food and beverage purchase” OR “food purchase” OR “food purchase data”)*

<u>Note</u>: A trained librarian drafted the search string for PubMed and translated across databases.

PART II

Single Attribute Results

Race/Ethnicity

Key findings from studies that presented racial/ethnic differences in consumer food and/or beverage purchases are summarized and presented by study in Table S1. Five studies examined *fruit, vegetable,* or *whole grain* purchasing [29, 39, 45, 46, 48], and three identified significant differences. Two identified greater fruit or fruit and vegetable purchasing identified among Hispanics compared to non-Hispanic Whites (henceforth NHW) [45, 46], one identified greater fruit and vegetable purchasing among Hispanics compared to non-Hispanic Blacks (henceforth NHB) [29], and Palmer et al. (2019) identified less fruit and vegetable purchasing among NHB compared to NHW [45]. Seven studies examined *salty snacks* and/or *desserts, sweet snacks, and candy* purchasing, and four identified significant differences [29, 30, 39, 45-48]. Paulin et al. (2001), Poti et al. (2016), and Palmer et al. (2019) identified less purchasing of salty snacks, desserts, and/or sweet snacks among Hispanics compared to NHW [45-47]. While Poti et al. (2016) and Palmer et al. (2019) identified less purchasing of these products among NHB compared to NHW [45, 47], Lenk et al. (2018) identified more purchasing of savory snacks among NHB compared to NHW [39]; which may relate to where customer purchasing was studied (i.e., limited-service versus full-service stores). Five studies examined *SSBs* or *non-sweetened beverage* purchasing [30, 31, 40, 47, 48]. Poti et al. (2016) identified significant differences with NHB purchasing more highly processed SSB and basic processed unsweetened fruit juice than NHW [47]. Stern et al. (2016) reported that SSB and fruit juices comprised a significantly greater proportion of total calories purchased among NHB compared to NHW and Hispanics [48].

Three studies examined purchasing using *HEI* and significant, though conflicting, results were identified [27, 53, 54]. Chrisinger et al. (2018) identified significantly better HEI-2010 scores among Hispanics than NHW [27]. Vadiveloo et al. 2019 also identified better HEI-2015 scores among Hispanic compared to NHW, but only in the southern region of the U.S. [53]. Among regions, they found either significantly worse scores or no significant differences. Vadiveloo et al. 2020 found that compared to NHW, NHB had significantly lower HEI-2015 scores, and NHO had higher scores, while there were no differences between NHW and Hispanic groups [54]. Five studies examined *kilocalories* purchased and all identified significant cross-sectional or longitudinal differences across racial/ethnic groups [29, 42, 47-49]. Despite these differences, the associations were inconsistent across studies. Some identified higher energy density purchasing among NHB compared to NHW [47-49]; others identified the opposite when examining overall kilocalories purchased) [42]. Among the nutrient purchasing outcomes, four studies examined *sugar* [43, 47-49], three examined *saturated fat* [47-49], and two studied *sodium* content [48, 49]. There was a consistent pattern that NHB had significantly higher purchasing of sugar than NHW across studies; whereas, findings on differences between NHW and other racial/ethnic groups were inconsistent. Two studies examining saturated fat identified significantly lower purchasing among Hispanics compared to NHW [47, 49], though differences for other groups were less clear. Stern et al. (2016) and Taillie et al. (2016) examined sodium density of food purchases and identified significantly higher purchasing among NHW compared to NHW across different food purchasing patterns and full-service food retail chains, respectively [48, 49].

Four studies examined purchasing outcomes that were not part of our primary outcomes of interest, including total grocery dollars spent and food and beverage purchases with *price*

promotions (e.g., coupons), *low-content nutrient claims* (e.g., reduced fat, no added sugar), *degree of processing* (e.g., highly-processed, minimally-processed), and *degree of ready-to-eat* (e.g., requires cooking, ready-to-heat) [29, 47, 48, 51]. All identified significant differences across racial/ethnic groups. Cullen et al. (2007) identified significantly greater grocery dollar spending on food and beverages among NHW than NHB or Hispanic. Across the other three studies [47, 50, 51], NHB, Hispanic, and non-Hispanic other (henceforth NHO) demonstrated less purchasing of food and beverage products with *price promotions* and *low-content nutrient claims* than NHW. In addition, Asians compared to NHW demonstrated less beverage purchasing with *low-content nutrient claims*, but more food purchases with *low-content nutrient claims* and food and beverage purchases with *price promotions*. *Degree of processing* and *ready-to-eat purchasing* also differed, with NHB and Hispanics demonstrating less purchasing of *highly processed* and *ready-to-eat* food products compared to NHW; however, NHB also purchased more *highly processed* beverage products.

Socioeconomic Status

Key findings from studies that evaluated socioeconomic differences in consumer food and/or beverage purchases are summarized here and presented by study in Table S2. Nine studies examined *fruit* and/or *vegetable* purchasing [26, 29, 31, 32, 34, 36, 39, 45, 49] with four identified significant differences [31, 34, 39, 45]. Greater purchases of fruit only, or fruit and vegetables were identified among higher SES groups as compared to lower SES groups. Four studies found no significant differences in vegetable purchasing only, or fruit and vegetables by SES groups [26, 29, 32, 34]. One study examined purchasing of *whole grains*, and did not find significant differences among SES groups [39].

Eleven studies examined *salty snacks* and/or *desserts, sweet snacks, and candy* purchasing [26, 29, 30-32, 34, 36, 37, 39, 45, 52]. One study reported only descriptive statistics of sweet snack purchases, across SES levels [36]. Of the nine studies reporting inferential statistics, three studies did not find any significant results across SES levels for these purchasing outcomes [26, 29, 39]. Seven studies that found significant results, but many inconsistent findings were noted. For example, Grummon et al. (2017) found a statistically significant relationship between SES and salty snacks purchasing, but French et al. (2014) did not [32, 34]. Nine studies examined *SSB* purchasing [26, 29, 30, 32-34, 36, 39, 52] and five examined *non-sweetened beverage* purchasing [29, 31, 34, 36, 52]. Of the studies that assessed SSB purchases, five reported significant differences among SES groups with most studies showing that higher SES groups had lower SSB purchasing or greater reductions in SSB purchasing over time. Among the studies that examined unsweetened beverage purchases, four studies reported significant findings, and three studies indicated that lower SES households purchase less unsweetened beverages [31, 34, 45], the fourth study was unable to identify the specific differences between the groups [29].

Three studies examined purchasing using *HEI* [28, 32, 54]. Overall, higher SES households had significantly higher HEI total scores than lower SES households. This finding was not consistent across SES groups for certain HEI sub-component scores. Five studies examined *kilocalories* with four studies identifying significant differences cross-sectionally or overtime across groups [30, 34, 42, 49, 52]. However, the variety of outcome measures used in these studies make synthesis of the results difficult. Two studies found that purchases among lower SES households were higher in energy density and total calories per person per day compared to higher SES

households [34, 49]. Another study found that low-income households purchased fewer calories per capita per day, but also had the slowest decrease in calories purchased per capita per day over time (2000-2013) [42].

Among the four papers that examined nutrient purchasing outcomes [34, 42, 49, 52], all four studies examined *sugar*, two examined *saturated fat* [49, 52], and three studied *sodium* content [34, 49, 52]. There was a consistent pattern that lower SES groups had higher purchases of sugar than higher SES groups regardless of measures (total sugar, added sugar, sugar density). Similarly, in the three studies that assessed sodium content of purchases, there was a consistent pattern that lower SES groups had higher purchases of sodium than higher SES groups. In the two studies that examined saturated fat content of purchases, one study did not find significant differences across SES groups [49], and the other found that the saturated fat content of purchases varied by store type for different SES groups [52].

Three studies examined purchasing outcomes that were not part of our primary outcomes of interest, so we classified the results of these studies into the *Other* category [29, 50, 51]. In two of these studies, SES was operationalized by using three groups (low, middle and high income groups) and both studies found significant differences across SES groups. In one study, high and middle income households had significantly higher proportions of purchases with *pricing promotions* compared to low-income households [51]. In the other study, high and middle income households had significantly higher proportions of purchases with *low-content nutrient claims* compared to low-income households [50]. The third study found no significant

differences in grocery dollar spending on food and beverages among differing levels of education [29].

Table S1. Key Findings from Studies Examining Racial/Ethnic Differences (n = 15)

**Authors (Year)**	**Racial/Ethnic Groups**	**Purchasing Outcomes Examined**										**Key Findings‡**
		F&V	WG	SS	Dess.	SSB	Bev	HEI	Kcals	Nutri.	Other	
Chrisinger (2018) [2]	NHW<br>NHB<br>Hisp<br>NHO							**X**				Hisp and NHO significantly higher **HEI-2010 scores** than NHW (ref). NHB not significantly different from NHW.
Cullen (2007)	NHW<br>NHB<br>Hisp	**X**		**X**	**X**	**X**	**X**				**X**	No significant between group differences identified for purchasing (percent of total grocery dollar spent on category) of **salty snacks**, **cakes/pies/ desserts**, **candy**, **carbonated and sweetened drinks**, **100% fruit juice**, and **water**. Hisp purchased a greater percentage of **fruit** and **vegetables** than NHB; NHW not significantly different from Hisp. **Other**: Total grocery dollars spent; NHW spent significantly more than NHB and Hisp.
Ford (2014)	NHW<br>NHB<br>Hisp			**X**	**X**	**X**			**X**			No significant differences across groups for change in purchases of **savory snacks, sweet snacks and candy, grain-based desserts, soda,** and **sweetened milk** (kcals/capita/day) from 2000-2011. Change (from 2000-2011) in **overall kcals** purchased was significantly less for Hisp (-233 kcal/capita/day) compared to NHW (-299 kcal/capita/day) and NHB (-296 kcal/capita/day).
Lenk (2018)	NHW<br>NHB<br>Combined[a]	**X**	**X**	**X**	**X**	**X**						Differences by groups examined in adjusted models only if significant in unadjusted (bivariate) models. UNADJUSTED: No significant differences across groups for purchasing at least 1 serving of **fruits and vegetables, whole grains**, **sweet baked goods**, or **candy**; significant differences identified for purchasing 1 serving of **savory snacks** and **SSB**. ADJUSTED: NHB and Combined more likely to purchase 1 serving of **savory snacks** compared to NHW (ref). No significant differences across groups identified for **SSB**.

Ng<br>(2016)	NHW<br>NHB<br>Hisp								X			Change in **overall kcals** (kcals/capita/day) from *food-only purchases* of consumer purchase goods (CPG) varied over time (2000-2013) across groups: at baseline (in 2000) NHB and Hisp were significantly lower than NHW (ref); from 2003-2006, the decline (observed among all groups) was significantly steeper for NHB (-66 kcal/capita/day) and Hisp (-61 kcal/capita/day) than NHW (-31 kcal/capita/day); however, from 2009-2012, decline accelerated for NHW (-109 kcal/capita/day) such that no significant difference was identified with Hisp (-88 kcal/capita/day) but a significant, slower decline difference identified for NHB (-66 kcal/capita/ day). For CPG *beverage-only purchases***:** No significant differences across groups at baseline or for change in **overall kcals** (2000-2013) except for the decline from 2009-2012 for NHB significantly slowed (-13 kcal/ capita/day) compared to NHW (-25 kcal/capita/day).
Ng<br>(2017)	NHW<br>NHB<br>Hisp<br>NHO									X		**Total sugars** (grams/capita/day) among *beverage-only purchases* in 2007/08, 2009/10, and 2011/12 were significantly higher among NHB and significantly lower among NHO and Hisp compared to NHW (ref). **Added sugars** (grams/capita/day) among *beverage-only purchases* in 2007/08, 2009/10, and 2011/12 were significantly higher among NHB and significantly lower among NHO compared to NHW; Hisp was only significantly lower than NHW in 2007/08 and not significantly different in 2009/10 and 2011/12.
Palmer<br>(2019)	NHW<br>Hisp<br>NHB<br>Asian<br>Another[b]	X		X	X							Compared to NHW (ref), smaller percentage of NHB purchased **fruit**, **vegetables**, and **snacks and sweets**. Compared to NHW, greater percentage of Hisp purchased **fruit** and smaller percentage of Hisp and Asian purchased **snacks and sweets**. No significant differences identified for Another.
Paulin<br>(2001)	NHW<br>Hisp[c]	X		X	X							Hisp significantly more weekly purchases of **fruits and vegetables** than NHW (ref). Hisp significantly less weekly purchases of **snack foods, pastry, and**

												**related items** (includes both salty and sweet items) than NHW.
Poti<br>(2016)	NHW<br>NHB<br>Hisp			**X**	**X**	**X**	**X**		**X**	**X**	**X**	In 2012, NHB and Hisp had significantly lower purchases than NHW (ref) for **highly-processed (HP) salty snacks, ready-to-eat (RTE) salty snacks, HP grain-based desserts, HP candy/ sweet snacks, HP dairy-based desserts, RTE grain-based desserts, RTE candy/ sweet snacks, RTE dairy-based desserts** (kcals/capita/day). In 2012, NHB had significantly higher purchases than NHW for **HP SSB** and **basic processed (unsweetened) fruit juice**; no significant differences between Hisp and NHW. **Energy density** (kcal/1000g) purchasing was significantly higher for NHB and significantly lower for Hisp compared to NHW. Purchases of **saturated fat** (% kcal) was significantly lower and **sugar** (% kcal) was significantly higher for NHB and Hisp compared to NHW. **Other**: Proportion of purchases (% of kcals) studied by 4 categories of degree of processing (**minimally-, basic-, moderately-** and **HP [highly-processed]**) and 3 categories of ready-to-eat (**requires cooking**, **ready-to-heat**, **RTE**).<br>Among *food-only purchases*, NHB and Hisp compared to NHW (ref) had significantly higher proportion of purchases for **basic-processed** and **requires cooking** categories and significantly lower proportion of purchases for **HP** and **RTE**; no other significant differences identified.<br>Among *beverage-only purchases*, NHB had significantly more **HP** and significantly lower **minimally-processed** purchases than NHW and Hisp not significantly different from NHW; no other significant differences identified.
Stern<br>(2016)	NHW<br>NHB<br>Hisp.	**X**		**X**	**X**	**X**	**X**		**X**	**X**		Across difference food-shopping patterns, the proportion of kcals purchased for **vegetables**, **salty snacks**, **grain-based desserts**, **candy**, and **sweet snacks** were similar across racial/ethnic groups. NHB has a greater proportion of calories from **SSB**

												and **fruit juices** than NHW and Hisp. Across food shopping patterns, NHB purchased packaged foods with significant higher **kcals**, **sugar density** (g/1000g), and **sodium density** (mg/1000g) than NHW and Hisp. No difference in **saturated fat** content were observed across groups. Across food shopping patterns, NHB purchased beverages with significantly higher **sugar density** (g/1000g) and lower **sodium density** (mg/1000g) than NHW and Hisp.
Taillie (2016)	NHW<br>NHB<br>Hisp.<br>NHO								**X**	**X**		Packaged food/beverage purchase outcomes examined by groups within retail chain type (Other food retail chains [FRC] versus Walmart) in 2000 and 2013. Among FRCs: **Energy density** (kcal/100g) was significantly greater among NHB and significantly lower among Hisp than NHW (ref) in 2000; no significant differences for NHO in 2000 or for any group in 2013. **Sugar density** (g/100g) was significantly greater among NHB in 2000 and 2013 and among NHO in 2013 than NHW; Hisp not significantly different from NHW. **Saturated fat density** (g/100g) was significantly lower among Hisp in 2000 and 2013 and among NHO in 2000 than NHW; NHB not significantly different from NHW. **Sodium density** (mg/100g) was significantly greater among NHB in 2000 and 2013 and among NHO in 2013 than NHW; Hisp not significantly different from NHW. Among Walmart: Results were either similar to FRC results or not significant, except for **saturated fat** which showed NHO was significantly higher than NHW in 2000.
Taillie (2017) [1]	NHW<br>NHB<br>Hisp.<br>NHO<br>Asian										**X**	Differences by groups examined in unadjusted models with statistical significance Bonferroni-corrected for multiple testing. **Other**: Proportion of transactions with a **low-content nutrient claim** (e.g., “low in,” “reduced,” “no”/”free of” nutrients, such as sugar, fat, sodium, and kcals) purchase pooled across 2008-2012. Among *packaged food-only purchases*, NHB, Hisp, and NHO had

												significantly lower and Asian significantly higher purchasing than NHW (ref). Among *packaged RTD beverage-only purchases*, NHB, Hisp, NHO, and Asian had significantly lower purchasing than NHW.
Taillie (2017) [2]	NHW<br>NHB<br>Hisp.<br>NHO<br>Asian										**X**	Differences by groups examined in unadjusted models with statistical significance Bonferroni-corrected for multiple testing. **Other**: Proportion of packaged purchases with a **price promotion** (i.e., any coupon or deal self-reported by households) pooled across 2008-2012. Among *packaged foods-only*, NHB, Hisp, and NHO had significantly lower and Asian significantly higher purchasing than NHW (ref). Among *packaged ready-to-drink (RTD) beverages-only*, NHB, Hisp, and NHO had significantly lower and Asian significantly higher purchasing than NHW.
Vadiveloo (2019)	NHW<br>NHB<br>Hisp.<br>NHO							**X**				**HEI-2015 scores** examined by groups within 4 US region. South region: Hisp significantly higher than NHW (ref); Midwest region: Hisp significantly lower than NHW; NHB and NHO not significantly different from NHW in South or Midwest. No significant differences across groups identified in Northeast or West regions.
Vadiveloo (2020)	NHW<br>NHB<br>Hisp.<br>NHO							**X**				Differences by group in **HEI-2015 scores** were examined in an UNADJUSTED ANALSIS. Compared to NHW (ref) HEI-2015 scores were lower among NHB, and higher among NHO. No significant differences were found between NHW and Hisp.

Note. NHW, non-Hispanic White; NHB, non-Hispanic Black; Hisp, Hispanic; NHO, non-Hispanic Other following author definition; Asian, authors definition of Asian; Combined, authors combined remaining race/ ethnicity groups; Another, authors definition of ‘Another race.’ F&V, fruits and/or vegetables; WG, whole grains; SS, Salty Snacks; Dess., desserts, sweet snacks & candy; SSB, sugar-sweetened beverages; Bev, non-sweetened beverages; HEI, healthy eating index; Kcals, kilocalories; Nutri., sugar, saturated fat, and/or sodium; Other, other purchasing outcomes examined; ref, reference group in modeling; HP, highly-processed; RTE, ready-to-eat; RTD, ready-to-drink; CPG, consumer package goods; FRC, other food retail chains; g, grams; mg, milligrams.

‡Findings present results from adjusted models unless otherwise noted. Significant results follow the authors’ definition (e.g., some use Bonferroni correction). Covariate information for adjusted models for each study is available in Supplemental Appendix. **Underline-bold** highlights purchasing outcomes of interest in this review. *Underline-italics* indicates when results for kilocalories/ energy density, sugar, saturated fat, sodium, or Other category was examined among food purchases and beverage purchases separately.

a) Authors combined participants that identified their race/ethnicity as Native American, Asian, Pacific Islander, Hispanic, and/or Other.
b) Authors did not specify which race and/or ethnicity were combined in the “Another race” category.
c) Authors limited analyses to participants that identified their race as White; comparisons are among White participants who identify as either non-Hispanic or Hispanic.

Table S2. Key Findings from Studies Examining Socioeconomic Differences (n = 19)

**Authors (Year)**	**SES Groups**	**Purchasing Outcomes Examined**										**Key Findings‡**
		F&V	WG	SS	Dess.	SSB	Bev	HEI	Kcals	Nutri.	Other	
Chrisinger (2018) [1][a]	WIC<br>Non-WIC	**X**		**X**	**X**	**X**						There were no significant differences in purchases by level of SES.
Chrisinger (2018) [2]	**Income level:**<br>SNAP<br>Non-SNAP El<br>SNAP InE:<br>(100-185% FPL)<br>SNAP InE,<br>(>185% FPL)<br><br>**Education Level:**<br><HS<br>HS/GED<br>Some college							**X**				SNAP had significantly lower **HEI-2010** scores than SNAP InE >185% FPL (ref). Non-SNAP El, and SNAP InE with incomes 100-185% FPL not significantly different than SNAP InE >185% FPL (ref). HS/GED had significantly lower **HEI-2010** scores than some college (ref). <HS not significantly different than some college (ref).
Cullen (2007) [x]	**Education Level:**<br><HS/HS<br>Some college<br>College degree<br>Advanced degree	**X**		**X**	**X**	**X**	**X**				**X**	No significant between group differences identified for purchasing (percent of total grocery dollar spent on category) of **fruit, vegetables, salty snacks, cakes/pies/ desserts**, **candy**, **carbonated and sweetened drinks**, **water,** and **total grocery dollars spent**. Significant differences were found between the three educations levels for 100% **juice** and **dairy** categories, however, these differences were not identified in post hoc analyses.
Ford (2014)	**Income level:**<br><130% FPL<br>130-185 % FPL<br>>185% FPL<br><br>**Education Level:**<br><HS<br>HS<br>College			**X**	**X**	**X**			**X**			No significant changes differences over time (2000-2011) across education level or income level group for **overall calories from food and beverages, or calories from savory snacks, sweet snacks and candy,** and **sweetened milk**.<br><br>Change (from 2000-2011) in **total beverage calories** was significantly less among <130% FPL, than the two higher income

												groups, and was significantly less among HS than <HS and college. Change (from 2000-2011) in calories from **soda** was significantly more among those who have <HS and 130-185% FPL than other groups. Change in calories from **milk** was greater among those with a college degree and 130-185% FPL than other groups. <130% FPL had the smallest decrease in **grain-based dessert** calories.
Frankle (2017)	SNAP<br>Non-SNAP	**X**		**X**	**X**	**X**	**X**					SNAP purchases were more likely to be for **sweet or salty snacks, candy, cold or frozen desserts, sweet bread, cakes or cookies and SSBs**, and less likely to be **for fruit, vegetables,** and **unsweetened beverages** compared to non-SNAP purchases.
French (2014)	LI (0 -1.3 IPR)<br>MI (1.4-3.4 IPR)<br>HI (>3.5 IPR)	**X**		**X**	**X**			**X**				HI had significantly higher **HEI-2010** composite scores than LI. LI spent more on **frozen desserts** than MI and HI. When looking at food groupings there were no differences in spending on **fruit, vegetables, or salty snacks** across income levels.
Gorski Findling (2018)	SNAP<br>Non-SNAP El<br>SNAP InE					**X**						SNAP InE spend a significantly smaller proportion of their overall food spending on **SSBs** than SNAP and Non-SNAP El, however, when looking at absolute SSB spending, SNAP InE spent significantly more than Non-SNAP El. There were no significant differences in absolute SSB spending between SNAP and SNAP InE or SNAP and Non-SNAP El. For adolescents' independent SSB purchases, SNAP spent a significantly higher proportion of their overall food spending on SSBs, but did have any significant differences in absolute spending on SSBs compared to Non-SNAP El and SNAP InE.
Grummon (2017)	SNAP<br>Non-SNAP El	**X**		**X**	**X**	**X**	**X**		**X**	**X**		Purchases of **fruit, salty snacks, SSBs,** and **total calories** (kcal/person/day), and **sugar**

	SNAP InE											and **sodium** (grams/day) were significantly different among Non-SNAP El and SNAP InE compared to SNAP (ref). Purchases of 100% fruit juice was significantly different between SNAP InE and SNAP (ref).<br>There were no significant differences in purchases of **vegetables, dessert and sweet snacks, candy and gum,** and **amount of saturated fat** in purchases.
Gustafson (2017)	SNAP<br>Non-SNAP El	**X**			**X**	**X**	**X**					Odds of purchasing **SSBs** and **unsweetened beverages** versus not purchasing these items (ref) for SNAP versus Non-SNAP El. SNAP and Non-SNAP El both have higher odds of purchasing SSBs (relative to not purchasing SSBs) when shopping at a supercenter, and convenience store. SNAP also had higher odds of purchasing SSBs at a grocery store. SNAP and Non-SNAP El both have higher odds of purchasing **water/low calorie beverages** at supercenters (relative to not purchasing water/low calorie beverages). SNAP also had higher odds of purchasing water/low calorie beverages at convenience stores.
Jones (2003)	LI Store<br>HI Store			**X**	**X**							UNADJUSTED ANALYSIS examined quantity shares (% of product class share within overall product category) and unit prices in LI and HI stores. HI stores had higher quantity shares of healthy **salty snacks**, and lower quantity shares on less healthy and regular salty snacks compared to LI stores. Quantity shares of **ice cream** across 6 levels of nutritional health were inconsistent.
Lenk (2018)	**Education level:**<br><HS/HS<br>Some college<br>College degree	**X**	**X**	**X**	**X**	**X**						**College degree** had increased odds of purchasing **F & V** compared to some college and <HS/HS. However, no differences were found in purchasing by education level for **whole grains, non/low-fat dairy, sweet baked goods, candy,** and

	**Employment Status:**<br>Employed<br>Unemployed											**savory snacks**. Unemployed had greater odds of buying **SSBs** than employed, no other significant differences were found in purchases by employment status.
Ng (2016)	LI ≤185% FPL<br>MI 186-400% FPL<br>HI ≥400% FPL								**X**			Calories of **CPG foods and beverages** purchased were highest among HI. Calories purchased declined for all income groups from 2000-2013. MI (-98 kcal/per capita/day) and LI (-96 kcal/ per capita/day) had a slower rate of decline in calories of CPG foods purchased compared to HI (-114 kcal/per capita/day) ($p < 0.01$).
Ng (2017)	LI ≤185% FPL<br>MI 186-400% FPL<br>HI ≥400% FPL									**X**		**Total sugars and added sugars** (grams/capita/day) among *beverage-only purchases* in 2007/08, 2009/10, and 2011/2012 were significantly higher along LI and MI compared to the HI (ref) at each time point.
Palmer (2018)	LI <200% FPL<br>HI ≥200% FPL	**X**		**X**	**X**							UNADJUSTED: A greater percentage of HI than LI purchased **fruit, vegetables, snacks** and **sweets.**
Taillie (2016)	LI <130% FPL<br>HI ≥130% FPL								**X**	**X**		Packaged food/beverage purchase outcomes examined by income groups within retail chain type (Other food retail chains [FRC] versus WalMart) in 2000 and 2013. **Energy density** (kcal/100g) was significantly greater among LI than HI (ref) in 2000 and 2013. **Sugar density** (g/100g) of WalMart purchases declined for both HI and LI from 2000-2013, with no significant differences in the amount of decline. In FRCs **sugar density** was significantly higher in LI than HI. **Sodium density** (mg/100g) of WalMart purchases for both LI and HI significantly declined from 2000-2013, with HI having a greater decline. **Saturated fat density** (g/100g) there were no significant differences in saturated fat by income group.

Taillie (2017) [1]	LI ≤135% FPL<br>MI 136-300% FPL<br>HI ≥300% FPL										X	Differences by groups examined in unadjusted models with statistical significance Bonferroni-corrected for multiple testing. **Other**: Proportion of transactions with a **low-content nutrient claim** (e.g., "low in," "reduced," "no"/"free of" nutrients, such as sugar, fat, sodium, and kcals) purchase pooled across 2008-2012. Among *packaged food and beverage purchases*, HI and MI had significantly higher proportions of purchases with claims then LI (ref).
Taillie (2017) [2]	LI ≤135% FPL<br>MI 136-300% FPL<br>HI ≥300% FPL										X	Differences by groups examined in unadjusted models with statistical significance Bonferroni-corrected for multiple testing. **Other**: Proportion of packaged purchases with a **price promotion** (i.e., any coupon or deal self-reported by households) pooled across 2008-2012. Among *packaged foods and ready-to-drink beverages*, HI and MI had a significantly higher proportion of purchases with price promotion than LI (ref).
Taillie (2018)	SNAP<br>Non-SNAP El<br>SNAP InE	X		X	X	X	X		X	X		Nutritional profile of packaged food/beverage purchase outcomes examined by store type across groups. **Fruit** SNAP InE significantly more likely to purchase fruit than SNAP at grocery stores and less likely than SNAP to purchase fruit at big box stores. **Vegetables** SNAP InE less likely to purchase non-starchy vegetables at big box stores than SNAP. Non-SNAP El less likely than SNAP to purchase starchy vegetables at grocery and big box stores. SNAP InE less likely than SNAP to purchase starchy vegetables at grocery and big box stores. **Junk food** Non-SNAP El and SNAP InE less likely to buy junk food than SNAP at grocery and big box stores. **Sweet snacks and desserts** Non-

												SNAP El and SNAP InE less likely to buy sweet snacks and desserts than SNAP at grocery and big box stores. <u>**Candy and gum**</u> SNAP InE more likely to buy candy and gum than SNAP at grocery stores only. <u>**SSBs**</u> Non-SNAP El and SNAP InE less likely to buy SSBs than SNAP at grocery, big box, and other stores. Non-SNAP El were also less likely to buy SSBs than SNAP at convenience stores. <u>**Calories**</u> Non-SNAP El and SNAP InE bought fewer kcals/capita/day than SNAP at grocery, big box, and convenience stores. SNAP InE also bought fewer kcals/capita/day than SNAP at other stores. <u>**Nutrients**</u> Non-SNAP El and SNAP InE bought fewer g/capita/day of sugar, saturated fat and sodium than SNAP at grocery and big box stores. Non-SNAP El bought fewer g/capita/day of sugar at convenience stores. SNAP InE bought fewer g/capita/day of sugar and sodium than SNAP at other stores.
Vadiveloo (2020)	SNAP<br>Non-SNAP<br>**IPR**<br><130% FPL<br>130-349 % FPL<br>>350% FPL							**X**				UNADJUSTED ANALYSES examined mean HEI-2015 scores by SNAP status and IPR. Non-SNAP participants had significantly higher scores compared to SNAP Participants. Also, the group with the highest IPR (>350%) had significantly higher scores than either lower IPR group.

Note. SES, socioeconomic status; F&V, fruits and/or vegetables; WG, whole grains; SS, Salty Snacks; Dess., desserts, sweet snacks & candy; SSB, sugar-sweetened beverages; Bev, non-sweetened beverages; HEI, healthy eating index; Kcals, kilocalories; Nutri., sugar, saturated fat, and/or sodium; Other, other product categories or nutrients examined; ref, reference group in modeling; HP, highly-processed; RTE, ready-to-eat; RTD, ready-to-drink; CPG, consumer package goods; FRC, other food retail chains; g, grams; mg, milligrams. WIC, Supplemental Nutrition Program for Women Infants and Children; Non-WIC, non-participants of WIC; SNAP, Supplemental Nutrition Assistance Program; Non-SNAP El, households who are income eligible for SNAP, but do not currently participate; SNAP InE, households who are not eligible for SNAP and do not participate; FPL, federal poverty level; <HS, less than a high school diploma; HS, high school; GED, graduate equivalency degree; IPR, income to poverty ratio; LI, low-income; MI, middle income; HI, high income.

‡Findings present results from adjusted models unless otherwise noted. Significant results follow the authors' definition (e.g., some use Bonferroni correction). Covariate information for adjusted models for each study is available in Supplemental Appendix. <u>**Underline-bold**</u> highlights purchasing outcomes of interest in this review. <u>*Underline-italics*</u> indicates when results for kilocalories/ energy density, sugar, saturated fat, sodium, or Other category was examined among food purchases and beverage purchases separately.

a) This paper presented findings from a targeted population of Black women.

PART III

Targeted Population Results

Single Factor Targeted Populations

Key findings from targeted studies are provided in Table S3 below. Six studies reported purchasing for a single factor targeted population [21, 22, 24, 25, 28, 41]. These populations were low-income individuals or households [21, 41] and individuals or households residing in an urban city [22, 24, 25, 28]. Studies with a low-income targeted population focused on participants of federal food assistance programs such as SNAP and the Special Supplemental Nutrition Program for Women, Infants, and Children (WIC). Four articles examined *fruit* and *vegetable* purchasing [22, 24, 25, 41] while one examined *whole grain* purchasing [25]. Appelhans et al. (2017) found that urban households purchased < 1 cup of fruit and vegetables per 1000 kilocalories [22]. Two studies by Caspi et al. (both published in 2017) reported that < 8% of urban customer purchases from limited-service stores were a fruit or vegetable item [24, 25]. Furthermore, Caspi et al. (2017) observed that < 8% of purchases included one serving of whole grains; most whole grain items purchased were salty snacks [25]. Two studies examined *salty snack*, *dessert*, *sweet snack*, and/or *candy* purchasing [24, 41]. Lin et al. 2014 found that 82% of low-income households purchased baked goods/sweets each week [41]. Caspi et al. 2017 observed that 15% and 13% of urban limit-service store customers purchased one or more serving of candy and sweet baked goods, respectively [24]. Two studies, Andreyeva et al. (2012) and Caspi et al. (2017) reported on *SSB* and *non-sweetened beverage* purchasing [21, 24]. Both concluded that SSB purchasing was high among study participants. Andreyeva et al. (2012) found that SNAP households purchased significantly greater volumes of SSBs, carbonated soft drinks, and fruit drinks from supermarkets compared to WIC-only households [21]. Three studies examined purchasing using *HEI* [22, 25, 28]. While Appelhans et al. (2017) and Crane et al.

(2019) reported mean HEI-2010 total scores of 59 among customer purchases (from all stores) [22, 25], Caspi et al. (2017) found a mean score of 39 among urban customer purchases from specifically limited-services (a score of 50 is considered average quality) [25]. Only two studies assessed *kilocalorie*, *saturated fat*, *sugar*, and/or *sodium* content [22, 24]. Again, both studies reported high volumes among customer purchases.

Table S3. Key Findings from Targeted Studies (N = 11)

**Authors (Year)**	**Target Population & Description**	**Purchasing Outcomes Examined**										**Key Findings‡**
		F&V	WG	SS	Dess.	SSB	Bev	HEI	Kcals	Nutri.	Other	
Andreyeva (2012)	Low-Income<br><br>(WIC & SNAP participants)					**X**	**X**					WIC + SNAP households had significantly higher expenditures and total beverage volume share for **SSB**, **carbonated soft drinks**, and **fruit drinks** compared to WIC only households (ref). WIC only households had significantly higher expenditures and total beverage volume share for **diet beverages** and **100% fruit juice** compared to WIC + SNAP households. WIC only households had significantly higher total beverage volume share for **unsweetened beverages** than WIC + SNAP households. No significant differences between groups for bottled water.
Appelhans (2017)	Urban<br><br>(Residents of an urban city)	**X**						**X**	**X**	**X**		**Whole fruit** purchases among households (cups/1000 kcal): median = 0.3, IQR = 0.1-0.6. **Vegetable** purchases (cups/1000 kcal): median = 0.6, IQR = 0.2-1.0. **HEI-2010 total score**: median = 59.4, IQR = 46.7-72.6. **Kcals** from food (kcal/g): median = 2.0, IQR = 1.6-2.6. Kcals from beverages: median = 0.4, IQR = 0.3-0.6. **Saturated fat** from purchases (g/1000 kcal): median = 12.8, IQR = 10.5-15.5. **Sugar** from purchases (g/1000 kcal): median = 54.6, IQR = 41.7-73.0. **Sodium** from purchases (mg/1000 kcal): median = 1252, IQR = 923-1755.
Borradaile (2009)	Low-Income + Urban<br><br>(Low-income children in an urban city)			**X**	**X**	**X**	**X**		**X**	**X**	**X**	**Chips** comprised 33.5% of all purchases among children. **Candy** represented 21.3% of purchases, **ice cream** 6.4%, and **pastries** 5.3% of purchases. About 88% of beverages purchased were **SSB**. About 45.7% were **fruit drinks,** 26.5% **soda**,

												12.1% tea/lemonade, and 10.9% **water**. On average, total **kcals** purchased per trip was 356.6 (± 290.3 kcal), **saturated fat** 3.8g (± 5.4), **sugars** 31.8g (± 35.8) and **sodium** 535.8mg (±777.2).
Caspi (2017) [1]	Urban<br><br>(Residents of an urban city)	**X**		**X**	**X**	**X**	**X**		**X**	**X**		About 2%, 6%, 17%, and 8% of participants purchased 1 or more servings of **fruit**, **vegetables**, **savory snacks**, and **artificially sweetened beverages**, respectively. No differences were detected by store type. About 15%, 13% and 46% of participants reported purchasing 1 or more servings of **candy**, **sweet baked goods**, and **SSB**, respectively. Significant differences detected by store type. Average **HEI-2010 total score** for purchases was 36.4. No significant difference detected by store type. Overall, median of **kcals** purchased was 540 (253-1287) and median % of kcal from **saturated fatty acids** was 6.2 (0-13). Significant differences were detected by store type.
Caspi (2017) [2]	Urban<br><br>(Residents of an urban city)	**X**	**X**					**X**				Among purchases, 8% included at least one **fruit** or **vegetable**. Increased amount (in pounds) of FV available in store and increase varieties of FV were significantly associated with greater odds of purchasing a fruit or vegetable. Among purchases, 8% included at least one serving of **whole grains** (most were snack items such as popcorn and tortilla chips). Pounds and varieties not associated with whole grain purchasing. Mean **HEI-2010 total score** for food purchases was 31 (±13). More store shelf space for fruits and beverages, higher healthy vs. unhealthy food ratio, and higher healthy food availability scores were associated with higher HEI-2010 purchase scores.

Chrisinger (2018) [1]	Black + Urban<br><br>(Black women in an urban city)	**X**		**X**	**X**	**X**						Overall, 14% of dollars spent were on **fruits & vegetables** (combined), 11% of dollars spent were on **sweet & salty snacks** (combined), and 6% of dollars spent were on **SSB**. Actual dollar amount was not reported. No significant differences detected by SES status.
Crane (2019)	Urban<br><br>(Residents of an urban city)							**X**				**HEI-2010 total scores** for purchases were 59.5 (±16.0) and 59.9 (±16.0) for women and men, respectively, with no significant differences identified between gender groups. All HEI-2010 component scores were similar between men and women except for whole grains. Women (4.5 ± 3.7) had a significantly higher component score for whole grains than men (3.1 ± 3.1).
Kiszko (2015)	Low-Income + Urban<br><br>(Residents of low-income community in an urban city)			**X**	**X**	**X**						Overall, 12.2% of all purchases were **regular potato chips** (13.1% among regular bodega shoppers vs. 10.36% among less frequent shoppers). 22.34% of all purchases were for **sweets** such as cookies, cakes, candy, and ice cream (21.4% among frequent shoppers vs. 24.3% among less frequent shoppers). 29.3% of all purchases were for **regular soda** or other **SSB** (30.5% among frequent shoppers vs. 26.7% among less frequent shoppers). Data on fruit and vegetable purchasing was collected but not reported.
Lent (2014)	Low-Income + Urban<br><br>(Residents of low-income community in an urban city)	**X**		**X**	**X**	**X**	**X**		**X**	**X**		Among all participants **fruits and vegetables** were found in 2.3% of all intercepts. **Chips**, **pastries**, **candy**, and **ice-cream** were found in 17.9, 10.1%, 7.9% and 3% of intercepts, respectively. **SSB**, **regular soda**, **fruit-flavored drinks**, **100% juice**, **sports drinks/flavored water**, and **plain water** were found in 63.3%, 32.2%, 21.1%, 4.7%, 2.7%, and 14.1% of intercepts, respectively. Mean **kcals** of purchases were

												666 (±1064.6) and mean **sugars** (g) of purchases was 66.2 (±113) and mean **sodium** (mg) was 921.1 (±4368.3).
Lin (2014)	Low-Income<br><br>(SNAP participants)	**X**			**X**							About 97% of households purchased **non-canned fruits and vegetables** (about $7.32 spent/week), and 87% of household purchased **canned fruits and vegetables** (about $3.50 spent/week). About 82% of households purchased **baked goods** (about $2.88 spent/week) and 83% purchased **sweets** (about $1.67 spent/week).
O'Malley (2013)	Low-Income + Urban<br><br>(Residents of low-income community in an urban city)	**X**		**X**	**X**	**X**	**X**					Of total purchases, 5% of intercepts included **fruit** and 0% included **vegetables**. 21.7% included **snack foods** (no specification), 13.3% included **candy**, and 1.7% included **ice cream**. 55% of intercepts included **beverages** (no specification between sweetened and unsweetened)

Note: SNAP, Supplemental Nutrition Assistance Program; WIC, Special Supplemental Nutrition Program for Women, Infants, and Children. F&V, fruits and/or vegetables; WG, whole grains; SS, Salty Snacks; Dess., desserts, sweet snacks & candy; SSB, sugar-sweetened beverages; Bev, non-sweetened beverages; HEI, healthy eating index; Kcals, kilocalories; Nutri., sugar, saturated fat, and/or sodium; Other, other purchasing outcomes of interest; ref, reference group in modeling; g, grams; mg, milligrams.

‡Findings present results from descriptive statistics or adjusted models unless otherwise noted. Significant results follow the authors' definition. **Underline-bold** highlights purchasing outcomes of interest in this review.

REFERENCES

1.) Wilson, M.M.; Reedy, J.; Krebs-Smith, S.M. American Diet Quality: Where It Is, Where It Is Heading, and What It Could Be. *J Acad Nutr Diet* **2016**, *116*, 302-310.

2.) 2015-2020 Dietary Guidelines: Chapter 2 – Current Eating Patterns in the United States. Available online: https://health.gov/our-work/food-nutrition/2015-2020-dietary-guidelines/guidelines/chapter-2/current-eating-patterns-in-the-united-states/ (accessed on 30 June 2020).

3.) Volpe, R., Okrent, A. Assessing the Healthfulness of Consumers' Grocery Purchases. U.S. Department of Agriculture, *Economic Research Service* **2012**, *EIB-102*. Available online: https://ageconsearch.umn.edu/record/262129/ (accessed on 13 March 2020).

4.) Bandy, L., Adhikari, V., Jebb, S., Rayner, M. The use of commercial food purchase data for public health nutrition research: A systematic review. *PloS One* **2019**, *14*, e0210192.

5.) Drewnowski, A., Rehm, C. D. Energy Intakes of US Children and Adults by Food Purchase Location and by Specific Food Source. *Nutr J* **2013**, *12*, 59.

6.) An, R., Maurer, G. Consumption of Sugar-Sweetened Beverages and Discretionary Foods Among US Adults by Purchase Location. *Eur J Clin Nutr* **2016**, *70*, 1396-1400.

7.) Walker, R., Keane, C. R., Burke, J. G. Disparities and access to healthy food in the United States: A review of food deserts literature. *Health Place* **2010**, *16*, 876-884.

8.) Zhang, F.F., Lui, J., Rehm, C. D., Wilde, P., Mande, J. R., Mozaffarian, D. Trends and Disparities in Diet Quality Among US Adults by Supplemental Nutrition Assistance Program Participation Status. *JAMA Netw Open* **2018**, *1*, e180237.

9.) Lui, J. H., Jones, S. J., Sun, H., Probst, J. C., Merchant, A. T., Cavicchia, P. Diet, physical activity, and sedentary behaviors as risk factors for childhood obesity: an urban and rural comparison. *Child Obes* **2012**, *8*, 440-448.

10.) Hiza, H. A. B., Casavale, K. O., Guenther, P. M., Davis, C. A. Diet Quality of Americans Differs by Age, Sex, Race/Ethnicity, Income, and Education Level. *J Acad Nutr Diet* **2013**, *113* 297-306.

11.) Bowleg, L. The Problem with the Phrase Women and Minorities: Intersectionality—an Important Theoretical Framework for Public Health. *Am J Public Health* **2012**, *102*, 1267-1273.

12.) Bauer, G. R. Incorporating Intersectionality Theory Into Population Health Research Methodology: Challenges and the Potential to Advance Health Equity. *Soc Sci Med* **2014**, *110*, 10-17.

13.) Abrams, J. A., Tabaac, A., Jung, S., Else-Quest, N. M. Considerations for employing intersectionality in qualitative health research. *Soc Sci Med* **2020**, *258,* 113138.

14.) Hartmann-Boyce, J., Bianchi, F., Piernas, C., Payne Riches, S., Frie, K., Nourse, R., Jebb, S. A. Grocery store interventions to change food purchasing behaviors: a systematic review of randomized controlled trials. *Am J Clin Nutr* **2018**, *107*, 1004–1016.

15.) Gittelsohn, J., Trude, A. C. B., Kim, H. Pricing Strategies to Encourage Availability, Purchase, and Consumption of Healthy Foods and Beverages: A Systematic Review. *Prev Chronic Dis* **2017**, *14*, E107.

16.) Liberato, S. C., Bailie, R., Brimblecombe, J. Nutrition interventions at point-of-sale to encourage healthier food purchasing: a systematic review. *BMC Public Health* **2014**, *14*, 919.

17.) Epstein, L. H., Jankowiak, N., Nederkoorn, C., Raynor, H. A., French, S. A., Finkelstein, E. Experimental research on the relation between food price changes and food-purchasing patterns: a targeted review. *Am J Clin Nutr* **2012**, *95*, 789-809.

18.) An, R. Effectiveness of subsidies in promoting healthy food purchases and consumption: a review of field experiments. *Public Health Nutr* **2013**, *16*, 1215-1228.

19.) Abeykoon, A. H., Engler-Stringer, R., Muhajarine, N. Health-related outcomes of new grocery store interventions: a systematic review. *Public Health Nutr* **2017**, *20*, 2236-2248.

20.) National Heart Lung and Blood Institute (NHBLI). Study Quality Assessment Tools. Available online: https://www.nhlbi.nih.gov/health-topics/study-quality-assessment-tools (accessed on 20 February 2020).

21.) Andreyeva, T., Luedicke, J., Henderson, K. E., Tripp, A. S. Grocery Store Beverage Choices by Participants in Federal Food Assistance and Nutrition Programs. *Am J Prev Med* **2012**, *43*, 411-418.

22.) Appelhans, B., French, S. A., Tangney, C. C., Powell, L. M., Wang, Y. To what extent do food purchases reflect shoppers' diet quality and nutrient intake? *Int J Behav Nutr Phys Act* **2017**, *14*, 46.

23.) Borradaile, K. E., Sherman, S., Vander Veur, S. S., McCoy, T., Sandoval, B., Nachmani, J., Karpyn, A., Foster, G. D. Snacking in Children: The Role of Urban Corner Stores. *Pediatrics* **2009**, *124*, 1293-1298.

24.) Caspi, C., Lenk, K., Pelletier, J. E., Barnes, T. L., Harnack, L., Erickson, D. J., Laska, M. N. Food and beverage purchases in corner stores, gas-marts, pharmacies and dollar stores. *Public Health Nutr* **2017**, *20*, 2587-2597.

25.) Caspi, C., Lenk, K., Pelletier, J. E., Barnes, T. L., Harnack, L., Erickson, D. J., Laska, M. N. Association between store food environment and customer purchases in small grocery stores, gas-marts, pharmacies and dollar stores. *Int J Behav Nutr Phys Act* **2017**, *14*, 76.

26.) Chrisinger, B. W., Isselman DiSantis, K., Hillier, A. E., Kumanyika, S. K. Family food purchases of high- and low-calorie foods in full-service supermarkets and other food retailers by Black women in an urban US setting. *Prev Med Rep* **2018**, *10*, 136-143.

27.) Chrisinger, B. W., Kallan, M. J., Whiteman, E. S., Hillier, A. E. Where do U.S. households purchase healthy foods? An analysis of food-at-home purchases across different types of retailers in a nationally representative dataset. *Prev Med* **2018**, *112*, 15-22.

28.) Crane, M. M, Tangney, C. C., French, S. A., Wang, Y., Appelhans, B. Gender Comparison of the Diet Quality and Sources of Food Purchases made by Urban Primary Household Food Purchasers. *J Nutr Educ Behav* **2019**, *51*, 199-204.

29.) Cullen, K., Baranowski, T., Watson, K., Nicklas, T., Fisher, J., O'Donnell, S., Baranowski, J., Islam, N., Missaghian, M. Food Category Purchases Vary by Households Education and Race/Ethnicity: Results from Grocery Receipts. *J Am Diet Assoc* **2007**, *107*, 1747-1752.

30.) Ford, C. N., Ng, S. W., Popkin, B. M. Are food and beverage purchases in households with preschoolers changing? A longitudinal analysis from 2000–2011. *Am J Prev Med* **2014**, *47*, 275-282.

31.) Franckle, R. L., Moran, A., Hou, T., Blue, D., Greene, B. A., Thorndike, A., Polacsek, M., Rimm, E. B. Transactions at a Northeastern Supermarket Chain: Differences by Supplemental Nutrition Assistance Program Use. *Am J Prev Med* **2017**, *53*, e131-e138.

32.) French, S. A., Tangney, C. C., Crane, M. M, Wang, Y., Appelhans, B. Nutrition quality of 1food purchases varies by household income: the SHoPPER study. *BMC Public Health* **2019**, *19*, 231.

33.) Gorski Findling, M. T., Wolfson, J. A., Rimm, E. B., Bleich, S. N. Differences in the Neighborhood Retail Food Environment and Obesity Among US Children and Adolescents by SNAP Participation. *Obesity* **2018**, *26*, 1063-1071.

34.) Grummon, A. H., Taillie, L. S. Nutritional Profile of Supplemental Nutrition Assistance Program Household Food and Beverage Purchases. *Am J Clin Nutr* **2017**, *105*, 1433-1442.

35.) Grummon, A. H., Taillie, L. S. Supplemental Nutrition Assistance Program participation and racial/ethnic disparities in food and beverage purchases: SNAP and Racial/Ethnic Disparities. *Public Health Nutr* **2018**, *21*, 3377-3385.

36.) Gustafson, A. Shopping pattern and food purchase differences among Supplemental Nutrition Assistance Program (SNAP) households and Non-supplemental Nutrition Assistance Program households in the United States. *Prev Med Rep* **2017**, *7*, 152-157.

37.) Jones E., Akbay, C., Roe, B., Chern, W. S. Analyses of Consumers' Dietary Behavior: An Application of the AIDS Model to Supermarket Scanner Data. *Agribusiness* **2003**, *19*, 203-221.

38.) Kisko, K., Cantor, J., Abrams, C., Ruddock, C., Moltzen, K., Devia, C., McFarline, B., Singh, H., Elbel, B. Corner store purchases in a low-income urban community in NYC. *J Community Health* **2015**, *40*, 1084-1090.

39.) Lenk, K. M., Caspi, C. E., Harnack, L., Laska, M. N. Customer characteristics and shopping patterns associated with healthy and unhealthy purchases at small and non-traditional food stores. *J Community Health* **2018**, *43*, 70-78.

40.) Lent, M. R., Vander Veur, S., Mallya, G., McCoy, T. A., Sanders, T. A., Colby, L., Rauchut Tewksbury, C., Lawman, H. G., Sandoval, B., Sherman, S., Wylie-Rosett, J., Foster, G. D. Corner store purchases made by adults, adolescents and children: items, nutritional characteristics and amount spent. *Public Health Nutr* **2014**, *18*, 1706-1712.

41.) Lin, B., Ver Ploeg, M., Kasteridis, P., Yen, S. T. The roles of food prices and food access in determining food purchases of low-income households. *J Policy Modeling* **2014**, *36*, 938-952.

42.) Ng, S. W., Poti, J. M., Popkin, B. M. Trends in racial/ethnic and income disparities in foods and beverages consumed and purchased from stores among US households with children, 2000–2013. *Am J Clin Nutr* **2016**, *104*, 750-759.

43.) Ng, S. W., Ostrowski, J. D., Li, K. Trends in added sugars from packaged beverages available and purchased by US households, 2007–2012. *Am J Clin Nutr* **2017**, *106*, 179-188.

44.) O'Malley, K., Gustat, J., Rice, J., Johnson, C. C. Feasibility of Increasing Access to Healthy Foods in Neighborhood Corner Stores. *J Community Health* **2013**, *38*, 741-749.

45.) Palmer, A., Bonanno, A., Clancy, K., Cho, C., Cleary, R., Lee, R. Enhancing understanding of food purchasing patterns in the Northeast US using multiple datasets. Renewable *Agriculture and Food Systems* **2019**, 1-15.

46.) Paulin, G. D. Variation in Food Purchases: A Study of Inter-Ethnic and Intra-Ethnic Group Patterns Involving the Hispanic Community. *Family and Consumer Sciences Research Journal* **2001**, *29*, 336-381.

47.) Poti, J. M., Mendez, M. A., Ng, S. W., Popkin, B. M. Highly Processed and Ready-to-Eat Packaged Food and Beverage Purchases Differ by Race/Ethnicity among US Households. *J Nutr* **2016**, *146*, 1722-1730.

48.) Stern, D., Poti, J. M., Ng, S. W., Robinson, W. R., Gordon-Larsen, P., Popkin, B. Where people shop is not associated with the nutrient quality of packaged foods for any racial-ethnic group in the United States. *Am J Clin Nutr* **2016**, *103*, 1125-1134.

49.) Taillie, L. S., Ng, S. W., Popkin, B. M. Walmart and Other Food Retail Chains Trends and Disparities in the Nutritional Profile of Packaged Food Purchases. *Am J Prev Med* **2016**, 50, 171-179.

50.) Taillie, L. S., Ng, S. W., Xue, Y., Busey, E., Harding, M. No Fat, No Sugar, No Salt . . . No Problem? Prevalence of "Low-Content" Nutrient Claims and Their Associations with the Nutritional Profile of Food and Beverage Purchases in the United States. *J Acad Nutr Diet* **2017**, *117*, 1366-1374.

51.) Taillie, L. S., Ng, S. W., Xue, Y., Harding, M. Deal or no deal? The prevalence and nutritional quality of price promotions among U.S. food and beverage purchases. *Appetite* **2017**, *117*, 365-372.

52.) Taillie, L. S., Grummon, A. H., Miles, D. R. Nutritional Profile of Purchases by Store Type: Disparities by Income and Food Program Participation. *Am J Prev Med* **2018**, *55*, 167-177.

53.) Vadiveloo, M., Perraud, E., Parker, H. W., Parekh, N. Geographic Differences in the Dietary Quality of Food Purchases among Participants in the Nationally Representative Food Acquisition and Purchase Survey (FoodAPS). *Nutrients* **2019**, *11*, 1233.

54.) Vadiveloo, M., Parker, H. W., Juul, F., Parekh, N. Sociodemographic Differences in the Dietary Quality of Food-at-Home Acquisitions and Purchases among Participants in the U.S. Nationally Representative Food Acquisition and Purchase Survey (FoodAPS). *Nutrients* **2020**, 12, 2354.

55.) Bleich, S. N., Vercammen, K. A., Wyatt Koma, J., Li, Z. Trends in Beverage Consumption Among Children and Adults, 2003-2014. *Obesity* **2018**, *26*, 432-441.

56.) Di Noia, J., Monica, D., Cullen, K. W., Pérez-Escamilla, R., Gray, H. L., Sikorskii, A. Differences in Fruit and Vegetable Intake by Race/Ethnicity and by Hispanic Origin and Nativity Among Women in the Special Supplemental Nutrition Program for Women, Infants, and Children, 2015. *Prev Chronic Dis* **2016**, *13,* E115.

57.) Bailey, Z. D., Krieger, N., Agenor, M., Graves, J., Linos, N., Bassett, M. T. Structural racism and health inequities in the USA: evidence and interventions. *Lancet* **2017**, *389*, 8-14.

58.) Thompson, C., Cummins, S., Brown, T., Kyle, R. Understanding Interactions With the Food Environment: An Exploration of Supermarket Food Shopping Routines in Deprived Neighbourhoods. *Health Place* **2013**, *19*, 116-123.

59.) Cohen, D. A. Obesity and the Built Environment: Changes in Environmental Cues Cause Energy Imbalances. *Int J Obes* **2008**, *32*, S137-S142.

60.) Cohen, D. A., Babey, S. H. Contextual Influences on Eating Behaviours: Heuristic Processing and Dietary Choices. *Obes Rev* **2012**, *13*, 766-779.

61.) Cohen, D. A., Collins, R., Hunter, G., Ghosh-Dastidar, B., Dubowitz, T. Store Impulse Marketing Strategies and Body Mass Index. *Am J Public Health* **2015**, *105*, 1446-1452.

62.) Andreyeva, T., Tripp, A. S., Schwartz, M. B. Dietary Quality of Americans by Supplemental Nutrition Assistance Program Participation Status: A Systematic Review. *Am J Prev Med* **2015**, *49*, 594-604.

63.) Fielding-Singh, P. A Taste of Inequality: Food's Symbolic Value across the Socioeconomic Spectrum. *Sociological Science* **2017**, *4*, 424-448.

64.) Gibbons, J., Barton, M. S. The Association of Minority Self-Rated Health with Black Versus White Gentrification. *J Urban Health* **2016**, *93*, 909-922.

65.) Woolf, S. H., Braveman, P. Where Health Disparities Begin: The Role Of Social And Economic Determinants—And Why Current Policies May Make Matters Worse. *Health Affairs* **2011**, *30*, 1852-1859.

66.) Garcia, M. C., Faul, M., Massetti, G., Thomas, C. C., Hong, Y., Bauer, U. E., Iademarco, M. F. Reducing Potentially Excess Deaths From the Five Leading Causes of Death in the Rural United States. *MMWR Surveill Summ* **2017**, *66*, 1-7.

67.) Pinard, C. A., Byker Shanks, C., Harden, S. M., Yaroch, A. L. An integrative literature review of small food store research across urban and rural communities in the U.S. *Prev Med Rep* **2016**, *3*, 324-332.

68.) Houghtaling, B., Serrano, E. L., Kraak, V. I., Harden, S. M., Davis, G. C. A Systematic Review of Factors That Influence Food Store Owner and Manager Decision Making and Ability or Willingness to Use Choice Architecture and Marketing Mix Strategies to Encourage Healthy Consumer Purchases in the United States, 2005-2017. *In J Behav Nutr Phys Act* **2019**, *16*, 5.

69.) Shikany, J., Carson, T. L., Hardy, C. M., Li, Y., Sterling, S., Hardy, S., Walker, C. M., Baskin, M. L. Assessment of the nutrition environment in rural counties in the Deep South. *J Nutr Sci* **2018**, *7*, e27.

70.) Byker Shanks, C., Ahmed, S., Smith, T., Houghtaling, B., Jenkins, M., Margetts, M., Schultz, D., Stephens, L. Availability, Price, and Quality of Fruits and Vegetables in 12 Rural Montana Counties, 2014. *Prev Chronic Dis* **2015**, *12*, 150158.
